# Super enhancer-mediated transcription of miR146a-5p drives M2 polarization during *Leishmania donovani* infection

**DOI:** 10.1371/journal.ppat.1009343

**Published:** 2021-02-25

**Authors:** Sonali Das, Sohitri Mukherjee, Nahid Ali

**Affiliations:** Infectious Diseases and Immunology Division, CSIR-Indian Institute of Chemical Biology, Kolkata, India; Instituto de Biociências - Universidade de São Paulo, BRAZIL

## Abstract

The outcome of *Leishmania donovani* infection depends upon the dynamic interchanges between M1 and M2 macrophages. Information of the involvement of microRNAs (miRNAs) and epigenetic modifiers in regulating macrophage plasticity during *L*. *donovani* infection is still elusive. Differential expression analysis of polarization-regulating miRNAs, revealed significant enrichment of miR146a-5p during *Leishmania donovani* infection. A sustained enrichment of miR146a-5p was observed in both infected bone marrow derived macrophages (BMDMs) and BALB/c mice organs. We found involvement of miR146a-5p in phagocytosis and survivability of parasites. Moreover, miR146a-5pgot enriched in interleukin 4- stimulated BMDMs, indicating its possible involvement in M2 polarization. Upon transfecting BMDMs with miRVANA anti-146a oligos, M2 markers (CCR7, YM-1, FIZZ-1, arginase-1, IL10 and IL4) and transcription factors (p-STAT6 and c/EBPβ) got depleted with concomitant augmentation of M1-polarizing transcription factors (p-STAT1, AP1 and IRF-1), miR146a target genes (TRAF6 and IRAK1), M1 cytokines (IL12 and TNFα), *i*NOS, nitric oxide, and nuclear translocation of phospho p-65 subunit. Neutralization of intracellular mature miR146a-5p pool in infected BALB/c mice lower organ parasite burden and expressions of M2 markers and IL10 with enrichment of M1 markers like *i*NOS and IL12. Additionally, we explored the novel role of super enhancer (SE), a cis-acting regulatory component, to enrich miR146a-5p expression during infection. Enhanced expression and nuclear retention of SE components like BET bromodomain 4 (BRD4) and p300 were found in infected BMDMs. Upon silencing BRD4, expressions of miR146a-5p and M2 markers were down regulated and TRAF6, IRAK1 and *i*NOS levels increased. STRING V.11 based predication and immune precipitation confirmed the strong interaction amongst BRD4, p300 and RNA pol II (RpbI). Chromatin immune precipitation studies suggested the recruitment of BRD4 at the enhancer loci of miR146a-5p gene during infection. Altogether, our findings revealed a novel role of BRD4/p300-depdendent super-enhancer in regulating miR146a expression during *L*. *donovani* infection which in turn mediates M2 polarization and immune-suppression.

## Author summary

Visceral leishmaniasis (VL), caused by protozoan parasites *Leishmania donovani*, is the most severe form of leishmaniasis and is highly lethal if left untreated. Major obstacle for successful therapy of VL originates from the life-long immune-suppression triggered in the post *kala-azar* dermal leishmaniasis (PKDL) patients during infection. Identification of molecular principles behind such immune-suppression will add success in VL therapeutics. *L*. *donovani* hijacks the host macrophages and converts them from pro-inflammatory M1 to immune-suppressive M2 type, which allows successful infection establishment. Herein, we explored the indispensable role of miRNA-146a-5p in conversion of M1 to M2 type during infection. Both *in vitro* and *in vivo* miRNA silencing established miR146a-5p as an imperative negative regulator ofM1 polarization. Computational analysis as well as immune precipitation based experiments authenticated that *L*. *donovani* induces super enhancer complex mediated transcriptional upregulation of miR146a-5p. BET bromodomain protein 4 (BRD4) forms this SE complex along with p300 histone acetyl transferase and RNA pol II. Silencing of BRD4 significantly abrogated miR146a-5p mediated M2 polarization. In short, our current findings established a previously unrecognized role of BRD4-depdendent super enhancers in orchestrating persistent transcription of macrophage miR146a-5p which in turn promotes M2 polarization during *L*. *donovani* infection.

## Introduction

Visceral leishmaniasis (VL), caused by *Leishmania donovani*, results in almost 0.5–1 million new infection cases worldwide **[1]**. Lack of a successful vaccine, toxicity of available anti-leishmanial therapeutics, emergence of resistant parasite strains, HIV/VL co-infection and development of post *kala-azar* dermal leishmaniasis (PKDL) are causing therapeutic failure cases in VL **[2]**. A major hurdle in the successful treatment of the infection is the induction of chronic immune suppression in patients **[3].** *L*. *donovani* establishes an immune-supressive infection by colonizing within mammalian macrophages and trans-differentiating from promastigote to amastigote stage**[4]**. Recent studies indicated that the final outcome of VL largely depends upon dynamic interchanges between classically activated (M1) macrophage, which has leishmanicidal properties, and alternatively activated (M2) macrophage which supports parasite survival **[5,6]**. In general, bone marrow derived macrophages (BMDMs), polarized by Th2 cytokines like interleukin 4 (IL4) or interleukin 13 (IL13) are termed as M2 macrophages **[7]**. *L*. *donovani* skews T_helper_ (Th) response towards the Th2 type which involves induction of macrophage arginase 1 (Arg1) and IL10, and prevents pro-inflammatory cytokines and nitric oxide (NO) generation **[6]**. Besides Arg1, M2 macrophages are also characterized by enrichment of surface markers like C-C Motif Chemokine Receptor 7 (CCR7), chitinase 3 like-3 (YM-1), resistin-like molecule alpha(FIZZ1) as well as transcription factors like phosphorylated signal transducer and activator of transcription 6 (pSTAT6), CCAAT/enhancer-binding protein beta (c/EBPβ) etc.**[7–11]**.

On the contrary, macrophages activated by toll like receptor (TLR) ligands like lipopolysaccharide (LPS) and interferon γ (IFN γ), are called M1 macrophages. They are characterized by enhanced antigen presenting properties (APCs) and secretion of pro-inflammatory cytokines like IL12, tumor necrosis factor α (TNF α) etc., which establish a critical link between these APCs and CD8^+^ T cells. CD8^+^T cells secrete IFN γ which in turn stimulates activation of some immune-activating transcription factors like Nuclear Factor κ beta (NF-κB), phospho-STAT1, Interferon Regulatory Factor 1 (IRF-1),Activator protein 1 (AP-1) etc. in M1-polarized macrophages **[12–14]**. These transcription factors in turn mediate activation of induced nitric oxide synthase (*i*NOS) in M1 macrophages. Earlier studies explored key role of NF-κB mediated activation of *i*NOS in culminating NO-mediated clearance of *L*. *donovani* from macrophages **[15–16]**.

Recently, small RNA sequencing identified differential enrichment of immune-regulatory microRNAs (miRNAs) in VL patient peripheral blood monocytes (PBMCs) and *L*. *donovani*—infected THP-1 cells **[17–19]**. **Muxel et al**. reported that *Leismania amazoniensis* infection alters TLR pathway mediators in infected macrophages via modulation of let-7e. These reports cumulatively indicated a strong correlation between macrophage miRNA and immune-suppression, but information on the miRNAs in fine-tuning the M1/M2 plasticity during *L*. *donovani* infection is still elusive.

We have currently undertaken differential expression analysis of immune-regulating miRNAs from *L*. *donovani* infected BMDMs, to understand consequence of infection upon global landscape of macrophage polarizing miRNAs. Upon obtaining significant enrichment of miR146a-5p, we further testified involvement of the same in stimulating M2 polarized macrophage during *L*. *donovani* infection in BMDMs and BALB/c mice. miR146a-5p, located in the second exon of the LOC285628 gene in the mouse chromosome number 11, negatively regulates the lipopolysaccharide (LPS)-stimulated toll like receptor 4 (TLR4) pathways by targeting TNF receptor associated factor 6 (TRAF6) and interleukin-1 receptor-associated kinase 1 (IRAK1), in RAW264.7 macrophages **[20]**. Small-RNA sequencing earlier showed enrichment of miR146a-5pin *L*. *major* infected human PBMCs **[17]**. Moreover, the miRNA been shown to regulate macrophage polarization via TGFβ as well as Notch-pathway dependent manner **[21,22]**. Till date role of miR146a in macrophage polarization regulation has been explored in the light ofSMAD7-TRAF6-TGFβ pathway regulation **[22]**. However, involvement of miR146a-5p mediated regulation of M2 polarization via targeting TRAF6-IRAK1-NF-κB axis during *L*. *donovani infection* is still poorly explored. We found that, *L*. *donovani* infection results in persistent enrichment of miR146a-5p, which subsequently skews the infected macrophages towards the M2 profile. We also validated indispensability of miR146a-5p for *in vivo* infections by neutralizing intracellular mature miR146a-5p pool of BALB/c mice during of *L*. *donovani* infection.

During endotoxic stimulations, persistent transcription of miR146a has been previously addressed which signified a spectacular transcriptional regulatory element formation, at upstream cis-acting regions of miRNAs-encoding gene called super-enhancers (SEs) [23–24]. These elements are characterized by their long length, strength of inducing persistent transcription, and occupancy by unique transcription factors like BET bromo domain proteins (BRDs)/ RNA pol II/ p300, and their histone modification patters (H3K27Ac). Therefore, we explored the involvement of BRD4 and p300 and investigated their nuclear retention and chromosomal accumulation upon enhancer domain of miR146a-5p gene. Altogether, our findings signify involvement of a unique SE complex formation, upstream of miR146a-5p gene loci in BMDMs during *L*. *donovani* infection, which drives immune suppressive M2 polarization of the macrophages.

## Materials and methods

### Ethics statement

All the animal experiment protocols were performed according to the guideline of the Committee for the Purpose of Control and Supervision on Experimental Animals (CPCSEA), Ministry of Environment and Forest, Government of India, and were approved(IICB/AEC/Meeting/Feb/2018/10) by the Animal Ethics Committee (147/1999/CPSCEA) of CSIR-Indian Institute of Chemical Biology (CSIR-IICB), India.

### Parasite culture and maintenance in hamsters

*L*. *donovani* (MHOM/IN/1983/AG83; ATCC repository number PRAR-413) amastigotes were isolated from the spleen of three month infected Syrian golden hamsters (*Mesocricetus auratus)* and transformed into promastigotes at 22°C using Schneider’s Drosophila medium (Sigma-Aldrich, US) supplemented with 10% fetal bovine serum (FBS) (Gibco, Thermo Scientific, USA), 100 U/ml penicillin, 100 μg/ml streptomycin (Gibco, Thermo Scientific, USA) **[25]**. Virulence of the parasite strains was maintained by passaging through 4–6 week old hamsters, which were also bred and maintained in the animal care facility of CSIR-IICB. Metacyclic promastigotes, enriched in ficoll gradient **[26,27]** were used throughout the study to infect BMDMs and animals.

### Differentiation and infection of bone marrow derived macrophages

Bone marrow derived macrophages (BMDMs) were isolated from femurs of 4–6 week old BALB/c mice and cultured in Dulbecco’s modified eagles media (Gibco, Thermo scientific, USA) supplemented with10 ng/ml recombinant murine CSF (R&D systems, USA), 10% FBS and 100 U/ml penicillin, 100 μg/ml streptomycin (Gibco, Thermo scientific, USA) for 7days at 37°C in a fully humidified CO_2_ incubator. 7 days after, fully differentiated BMDMs were stimulated using lipopolysaccharide (LPS) (100 ng/ml, Invivogen, US) and interferon-γ (IFN-γ, 20ng/mL, eBioscience, San Diego, CA) for M1 macrophage activation. Stimulation with IL4 (20ng/mL, eBioscience, San Diego, CA) led to M2 macrophage activation and media alone was used for naïve (M0) macrophages **[7]**. Naïve BMDMs were infected with metacyclic promastigotes at 1:10 multiplicity of infection (MOI) ratio. For infection synchronization, extracellular parasites were washed with warm 20 mM phosphate buffered saline (PBS) and macrophages were incubated for the indicated times for infectivity related experiments.

### Animal infections

For *in vivo* infections, 2 X 10^7^ metacyclic promastigotes/ animal was suspended in 200 μl sterile 1.8% D-glucose infused 20 mM PBS and injected in 4–6 week old BALB/c mice via tail vein. In case of PBS control animals, 200 μl PBS was injected.

### Transfection of esiRNA pool and miRVANA miRNA inhibitor *in vitro*

Control scrambled RNA (scrRNA) (Sigma-aldrich, US) (25 pM/ml) and MISSION esiRNA for mouse BRD4 exons (60 pM/ml) (Sigma-aldrich, US) were transfected in BMDMs using Lipofectamine 2000 (Thermo Fischer, USA) following manufacturer’s protocol. For miRNA silencing, 20 nM mirVana miRNA inhibitor negative Control (anti-NC) and 40 nM antago miR named mirVana anti-miR-146a-5p inhibitor (anti-146a) were transfected in BMDMs using Lipofectamine 2000. Infection related studies were performed 24 hrs post transfection.

### *In vivo* neutralization of miR146a in animals

For *in vivo* neutralization of mature miR146a-5p, 4–6 weeks old BALB/c mice were infected with 2X10^7^ *L*. *donovani* suspended in 1.8% glucose- phosphate buffered saline solution. 28-days post-infection, synchronized infection was ensured in each group by Leishman Donovan unit (LDU) and limiting dilution assay (LDA) of spleen and liver. 25 μg/g mirVana miRNA inhibitor (0.025mg/g body weight) negative Control or antagomirs named mirVana anti-miR-146a-5p inhibitor oligo **[28]** were injected via tail vein in two separate groups (n = 3) of mice. On same day, a group of healthy age-matched littermates were injected with 200μl PBS via tail vein (n = 3). 7 days post neutralization, animals were euthanized for experiments.

### Parasite burden determination in animals

Spleen and liver were isolated from three month infected animals and parasite burden was determined by Leishman Donovan Unit (LDU) and limiting dilution assay (LDA). For LDU, stamp smears of organs were fixed with methanol and stained with Geimsa (Sigma Aldrich, US). LDU was represented by the number of amastigotes calculated in nearly 1000 macrophages from each slide of organ impressions multiplied by the respective organ weight in milligram (mg).

For LDA calculation, a 1 mg/ml (w/v) organ suspension (spleen or liver) prepared in Schneider drosophila media was serially diluted and cultured at 22°C for 2 weeks. Parasite burden was expressed as 10-fold logarithm scale of the highest dilution containing viable parasites, and the mean values of three mice per group were represented.

### Parasite phagocytosis rate determination

To determine the rate of phagocytosis, 5 X 10^7^*L*. *donovani* promastigotes were suspended in 1 ml PBS containing 1 μl of carboxyfluorescein diacetate succinimidyl ester (CFSE)(Molecular Probes, Thermo scientific, USA) from 2.8 μg/ ml dimethyl sulfoxide (DMSO, Sigma Aldrich, US) stock and incubated in 37°C humidified chamber for 10 min in the dark. CFSE stained parasites were allowed to interact with BMDMs for 4 hrs, unbound parasites were washed with 20 mM warm PBS and infected BMDMs were stained with anti-mouse Cd11b antibody-alexa fluor 700 (AF700, eBiosceinces, Thermo scientific, USA). Phagocytosis of zymosan particle by uninfected BMDM was used as positive control for the assay. A simultaneous set of healthy BMDMs were incubated with pHrodo green zymosan particle (Thermo scientific, USA) for 1 hr, followed by washing off the extracellular particle with PBS. CFSE signal of the phagocytized parasites and green fluorescent signal of up taken pHrodo zymosan particles from Cd11b^+^ cells were recorded using BD LSR-Fortessa flow cytometer (BD Biosciences, US). Data were analyzed using FACS Diva software.

### Small RNA extraction, reverse transcription and miRNA assay

Small RNA from BMDMs (4 X 10^7^) as well as organs (1mg) was isolated using miRVana miRNA isolation kit (Ambion, US) following manufacturer’s instruction. The cDNA pools of U6 small nuclear RNA and miR146a-5p were selectively produced from mature small RNAs using Taqman cDNA synthesis kit (Thermo scientific, USA), according to manufacturer’s protocol and the concentration was measured using Nanodrop 2000 (Thermo Scientific, USA). Briefly 100 ng pure small RNA was mixed with 5 X stem loop miRNA specific primer, 10 X RT buffer, dNTP mix w/dTTp, RNase inhibitor (20U/μL), and MultiScribe RT enzyme (50U/μL) in 25 μl reaction volume and incubated at 16°C and 42°C respectively for 30 mins followed by inactivation of enzyme at 85°C for 5 mins. After diluting the cDNA 25 times, 1.33 μl of cDNA solution was mixed with 20 X probed small RNA assay primers (miR-181a-5p, miR-146a-5p, miR-125a-5p, miR26a-5p and U6 snRNA), 2X Taqman universal master mix II (no UNG), and nuclease free water. The enzyme was activated at 95°C for 10 mins and denaturation of cDNA was done at same temperature for 15 sec followed by annealing and extension at 60°C for 60 sec respectively, for all the miRNAs. Amplification curves were obtained up to 40 cycles followed by holding the reaction at 4°C in real-time PCR (RT-PCR) (Light Cycler 96, Roche life sciences, Germany). Ct values for all the miRNAs and U6 snRNA were analyzed using Light cycler 96 software and fold changes were calculated with respect to U6 snRNA as reference gene using the following formula:

$$Fold\;change=2^{–\Delta \Delta Ct},\;\Delta \Delta Ct=\left(\Delta Ct_{experiment}‐\Delta Ct_{control}\right),\;\Delta Ct=\left(Ct_{miRNA}‐Ct_{U6\;snRNA}\right)$$

All the experiments were performed in triplicates. A negative control containing all reaction components except the reverse transcriptase enzyme was included and subjected to RT-PCR to confirm the absence of DNA contamination in RNA samples. List of primers used for PCR is provided in S1 Table.

### Total RNA extraction, reverse transcription and RT-PCR for mRNA expression

Total RNA was isolated from 4 × 10^7^ cells or 1 mg organ (in presence of liquid nitrogen) of different groups, using TriZOL-chloroform method (Ambion, Thermo scientific, USA). Pure RNA pellet was suspended in 50 μl di-ethyl pyrocarbonate (DEPC) treated water and the concentration was measured using Nanodrop 2000 (Thermo Scientific, USA). Almost 2 μg pure RNA was reverse transcribed using *i*Script cDNA synthesis kit (Biorad) following the manufacturer’s protocol. In brief, 2 μg RNA from each group, 5X *i*Script reaction mix, *i*Script reverse transcriptase enzyme and nuclease free water were added in 20 μl reaction volume, and the mixture was primed at 25°C for 5 mins, reverse transcribed at 46°C for 20 mins and heat inactivated at 95°C for 1 min. For RT-PCR, 2 μl cDNA, 0.5 μl 25 nM primer pairs of respective gene as well as internal control, 5 μl SYBR green master mix (Roche life sciences, Germany) and nuclease free water were added in 10 μl reaction mixture and PCR was performed in Roche Light Cycler 96.Ct values for control and test genes were analyzed using Light cycler 96 software and fold change was calculated using the following formula:

$$Fold\;change=2^{–\Delta \Delta Ct},\;\Delta \Delta Ct=\left(\Delta Ct_{experiment}‐\Delta Ct_{control}\right),\;\Delta Ct=\left(Ct_{gene}‐Ct_{Actin}\right).$$

All the experiments were performed in triplicates and mouse β-actin was used as reference gene. Details of primers are provided in S1 Table. Anegative control containing all reaction components except the reverse transcriptase enzyme was included and subjected to RT-PCR to confirm the absence of DNA contamination in RNA samples.

### Western blot

2 X 10^7^ BMDMs were washed with PBS and lysed in cell lysis buffer (150 mM sodium chloride, 1.0% Triton X-100, 0.5% sodium deoxycholate, 0.1% sodium dodecyl sulfate (SDS), 10 mM Di-thiothretol (DTT), 50 mM Tris, pH 8.0 containing protease and phosphatase inhibitor cocktail, Roche), and the protein concentrations in the cleared supernatants were estimated using Bradford method. The cell lysates were resolved on 10% SDS-PAGE and then transferred to nitrocellulose membranes (BioRad, US). The membranes were blocked with 5% Bovine Serum Albumin (BSA) (Sigma Aldrich, US) in Tris-buffered saline (TBS) for 1 hr at room temperature and probed with primary antibodies overnight at 4°C. Membranes were then washed three times with wash buffer (TBS containing 0.5% tween) and then incubated with HRP-conjugated secondary antibodies (Sigma Aldrich) and detected by Luminata Forte (Millipore Sigma, US) western HRP substrate (Fischer scientific, US). Densitometry data was analyzed using Image lab software version 5.0 (Biorad). The uncropped blots are provided in S2 Fig and the details of the antibodies are provided below-

Phospho NF-kB p65 (S536) (ab86299) mAb, IRAK1 Rabbit mAb (D51G7),BRD4 (E2A7X) Rabbit mAb,p300 (D8Z4E) Rabbit mAb, RabbitRpb1 mAb (D8L4Y), c/EBPβ (#3082), phospho-STAT sampler kit, DC-SIGN mAb (D7F5C) and Histone H3 (D1H2) XP Rabbit mAb were procured from Cell Signaling Technology (CST, US). Mouse TRAF6 (D-10) antibody and mouse IRF1 (E-4) were procured from Santa Cruz Biotechnology (SCBT, US). Anti-Siglec-E antibody (R&D systems, MN, USA) was kindly gifted by Prof. Chitra Mandal, CSIR-IICB, Kolkata. Rabbit AP1 mAb and β Actin monoclonal antibody, HRP conjugated anti-rabbit antibody and HRP conjugated anti-mouse antibody were procured from Sigma Aldrich.

### Immuno fluorescence confocal microscopy

2 X 10^5^ BMDMs were cultured on glass cover slips and infected with parasites at 1:10 MOI ratio for 4 hrs followed by washing with 20 mM PBS and fixation using 4% paraformaldehyde (Sigma Aldrich, US) for 10 mins at room temperature. After washing the excess fixative, cells were permeabilized using 0.25% Triton-X 100 (Sigma Aldrich, US) in PBS for 20 mins, followed by washing and blocking with 1% BSA and 22.52 mg/mL glycine in PBST (PBS with 0.1% Tween 20) for 30 mins. Then cells were incubated overnight with primary antibodies, (CST, US) diluted 1:200 times in blocking solution, at 4°C in a humidified chamber, followed by washing three times in PBST and incubation in the presence of secondary anti-rabbit-alexa fluor 488(Molecular Probes, Thermo Scientific, USA)and anti-mouse texus red (SCBT, US) (1:500 dilution) for 45 mins. Excess secondary antibodies were washed with PBS vigorously and nuclei were stained with Hoescht 33342 (Molecular probes, Thermo scientific, USA) for 10 mins in the dark. Coverslips were then mounted using 10% glycerol in PBS. Imaging was done in Leica TCS-SP8 confocal microscope and co-localization analysis of p-p65 with nuclear signal was done using LAS X software (Leica systems).

### Splenocyte isolation and culture from BALB/c mice

Splenocytes were isolated from infected and PBS, anti-NC and anti-miR146a-5p injected BALB/c mice and cultured in 10 μg/ml *Leishmania* Antigen (LAg)-supplemented RPMI media with 10% FBS and 100 U/ml penicillin, 100 μg/ml streptomycin for 24 hrs at 37°C in a fully humidified CO_2_ incubator. Supernatants of the cultured splenocytes were collected for ELISA.

### Cytokine Sandwich ELISA

Titers of IL12, TNFα and IL10 cytokines in differentially treated-BMDMs culture supernatants were determined by mouse Ready- SET- Go ELISA kits (eBiosciences, Thermo scientific, USA) following the manufacturer’s instruction. Sensitivity limit is 30 pg/ml for IL10, 8 pg/ml for TNFα and 2 pg/ml for IL12. Experiments were performed in triplicates and data were analyzed using graph pad prism version 5.0.

### Multicolor flow cytometry

2 X 10^6^ BMDMs were grown on 35 mm tissue culture dish and infected with *L*. *donovani* for 4 hrs, followed by warm 20 mM PBS wash and incubated for 18 hrs before blocking cytokine secretion by 10 μg/ ml Brefeldin A (5mg/ml) (BFA) (MP Biomedicals, US). 2 hrs after BFA treatment, cells are washed with chilled PBS three times and blocked with anti-mouse FcR antibody (CD16/CD32, BD) for 20 min at 4°C in presence of FACS buffer (20 mM PBS with 1% FBS). After washing the excess blocking antibody solution, cells were stained with anti-mouse Cd11b- fluorescein isothiocyanate labeled (FITC) antibody (1:100 dilution) (BD biosciences, US) for 1 hr at 4°C in a dark humidified chamber. After surface marker staining, cells were washed with PBS and treated with Cytofix/ Cytoperm (BD biosciences, US) for 20 mins at 4°C in the dark, followed by washing with FACS buffer containing 0.1% saponin (Sigma Aldrich). Cells were incubated with anti-mouse IL10-allophycocyanin (APC) and anti-mouse IL12-phycoerythrin (PE) (1:100 dilutions) for 1 hr at4°C in dark humidified chamber. After three times PBS washes, cells were procured for flow cytometry analysis in BD LSR-Fortessa flow cytometer (BD Biosciences, US). Experiments were done in triplicate and during acquisition of signal, gating of macrophage population was performed using auto polygon gating (Using BD FACSDiva software) on the basis of unstained compensated population. Then after doublet discrimination, each population was further analyzed for cytokine expressions. The final populations were represented as histogram plots denoting mean fluorescence intensity (MFI) for each cytokine.

### Immuno pull down assay and western blotting

For co-immunoprecipitation assay, 5X10^6^ BMDMs were lysed using RIPA lysis buffer supplemented with protease inhibitor cocktail (Sigma Aldrich), followed by denaturation of the lysate. After incubating the whole cell lysate with antibodies overnight at 4°C, desired protein were pulled down using protein A agarose beads (Sigma Aldrich). Pulled down protein is then resolved by SDS-PAGE and immune blotted using nitrocellulose membrane transfer method. The immune reactive bands were developed using Luminata Forte (Millipore Sigma, US) western HRP substrate (Fischer scientific, US). Densitometry data was analyzed using Image lab software version 5.0 (Biorad).

### Chemical cross-linked chromatin immune-precipitation, semi-quantitative and RT-PCR

Chromatins of 10X10^6^BMDMs were chemically cross-linked by drop-wise addition of 0.75% formaldehyde directly to the culture media and rotation for 10 mins gently at room temperature. The denaturant was quenched by adding 125 mM glycine and incubated for 5 mins followed by rinsing and scraping the cells with cold PBS. Cells were pelleted and lysed using FA lysis buffer (50 mM HEPES-KOH pH7.5, 140 mM NaCl, 1 mM EDTA pH8, 1% Triton X-100, 0.1% Sodium Deoxycholate, 0.1% SDS and Protease Inhibitors). Chromatin DNA present in the lysate was then fragmented in 250–500 bp fragments by 10 sonication cycles of 15 sec pulse (50% amplitude) and 45 sec interval for 10 mins. Protein A agarose beads pre-coated with 75 ng/ μl salmon sperm DNA (Thermo scientific) were incubated with antibodies of interest overnight at 4°C.After washing off excess unbound antibodies, chromatin containing supernatant was mixed with the bead mixture and incubated for 4–6 hrs at 4°C, followed by washing with gradient salt solution (as mentioned by Cross-linking Chromatin Immuno-precipitation protocol of Abcam). Finally bound DNA was eluted using 1% SDS, 100mM NaHCO_3_, treated with 0.5 mg/ml RNase A solution (Qiagen) and purified by PCR clean up kit (Qiagen). Almost 10 ng antibody pulled DNA and total input DNA were subjected to PCR experiments. The primers used for the amplification of 250 bp segment of miR146a-5p gene enhancer sequence, are mentioned in S1 Table. In short, 10 ng template, 25 nM forward and reverse primers, 2X DreamTaq PCR master mix (Thermo scientific), and nuclease-free water were mixed and the mixture was subjected to semi-quantitative PCR reaction of 95°C initial denaturation for 1 min, 25 cycles of 95°C denaturation for 30 sec, 48°C annealing for 30 sec, 72°C extension for 1 min and 72°C final extension for 7 mins. The PCR product was resolved on 1.5% agarose gel. For RT-PCR reaction,10 ng DNA, 0.5 μl 25 nM primer pairs of, 5 μl SYBR green master mix (Roche life sciences, Germany) and nuclease free water were added in 10 μl reaction mixture and PCR was performed in Roche Light Cycler 96.Ct values for IP DNA and input DNA were analyzed using Light cycler 96 software. Percentage of BRD4 accumulation upon miR146a-5p enhancer DNA was calculated by percent input method. 1% of initial input was used for ChIP, so a dilution factor (DF) of 100 or 6.644 cycles (i.e., log2 of 100) was subtracted from the Ct value of diluted input.

AdjustedinputCt=(RawinputCt‐6.644);Percentinput=100*2^(AdjinputCt‐IPDNACt)

Experiments were performed in triplicates and data were analyzed using graph pad prism version 5.0.

### Nuclear and cytosolic fractionation

Nuclear and cytosolic fractions were isolated from 5X10^6^BMDMs. Cell were initially lysed by incubating cells in nuclear buffer A (10 mM HEPES, 1.5 mM MgCl2, 10 mM KCl, 0.5 mM DTT, 0.05% NP40 pH 7.9), supplemented with protease inhibitor cocktail, for 10 mins at 4°C. The supernatant used was the cytosolic fraction and the pellet was further homogenized in the presence of nuclear buffer B (5 mM HEPES, 1.5 mM MgCl2, 0.2 mM EDTA, 0.5 mM DTT and 26% glycerol (v/v), pH 7.9). Following homogenization, the suspension was incubated for 30 mins at 4°C and centrifuged at 24,000 g for 30 mins. The supernatant was collected as the nuclear fraction and the protein content was measured by Bradford assay before western blotting.

### Quantification of nitric oxide level

Nitric oxide generated in culture supernatant of different groups of BMDMs was measured by Griess assay. Briefly 100 μl culture supernatant was mixed with 100 μl of Griess reagent (1% sulfanilamide and 0.1% N-(1-naphthyl) ethylene diaminedi- hydrochloride in 2.5% H_3_PO_4_) and incubated at room temperature for 10 min. Absorbance at 540 nm was then measured. 1 mM NaNO_3_ (Sigma Aldrich) solution prepared in media was used for standard curve generation. The graph for nitric oxide was generated using graph pad prism version 5.0.

### Statistical analysis

All the experiments were performed in biological and experimental triplicates. Statistical analyses were performed with one way ANOVA using the Graph Pad Prism Software version 5 (Graph Pad Software Inc., La Jolla, CA, USA). Significant differences were set at *P<0.05, **P<0.01, ***P<0.001. Data points represent error bar showing mean ± SD (standard deviation).

## Results

### *L*. *donovani* infection stimulates host miR146a-5p expression both *in vitro* and *in vivo*

**Fig 1:**
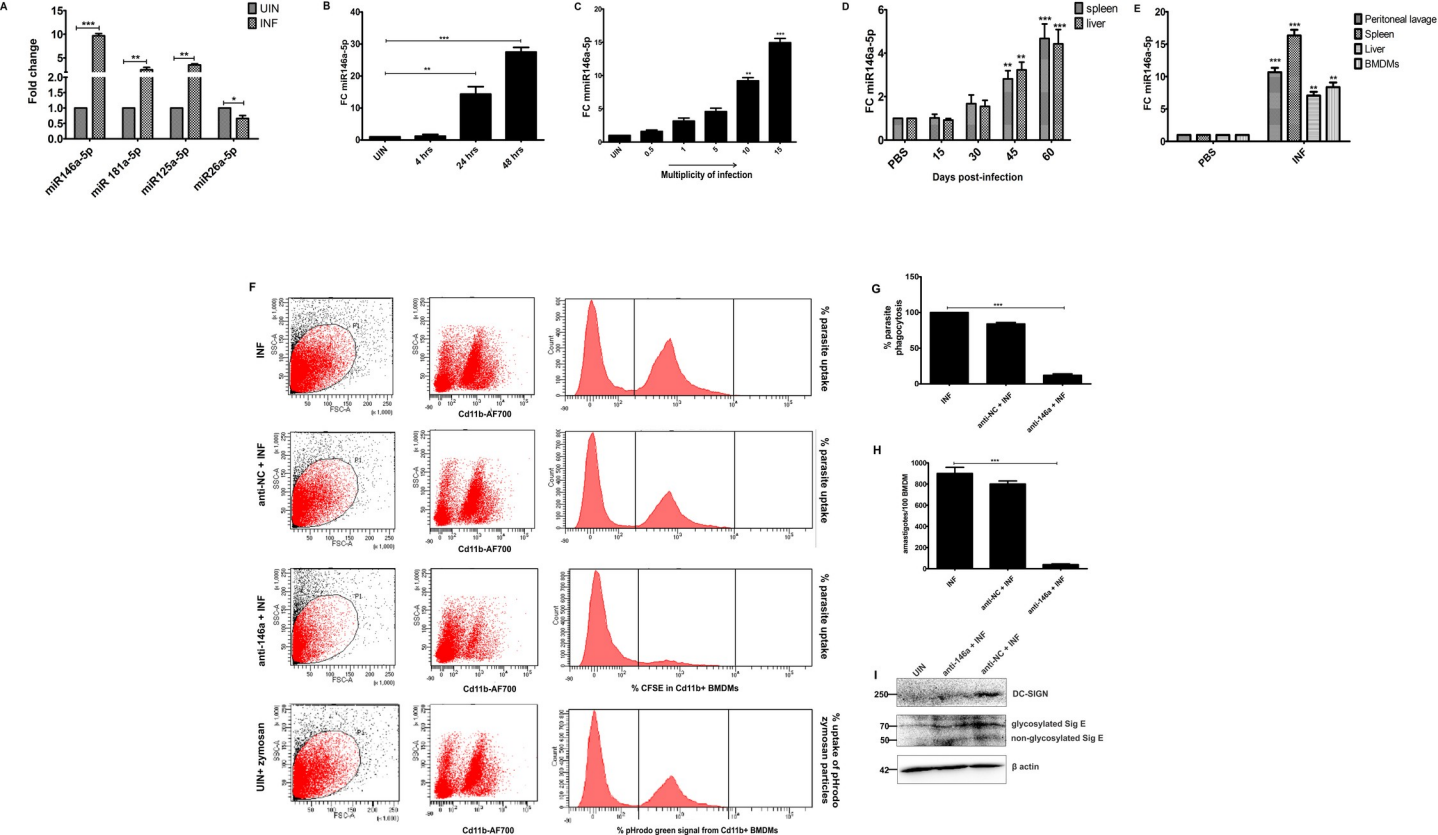
M2 polarizing miRNAs enrich in *L*. *donovani* infected BMDMs where miR146a-5p has an essential role in parasite phagocytosis and survival. **(A)** BMDMs were infected with *L*. *donovani* for 24 hrs and differential expression analysis of M1 (miR26a-5p and miR125a-5p) and M2 (miR146a-5p and miR181a-5p) polarization regulating miRNAs were performed by RT-PCR. RT-PCR based evaluation of mature miR146a-5p expression in *L*. *donovani* infected BMDMs using either **(B)** using 1:10 multiplicity of infection ratio (MOI) for different time points or **(C)** using different MOIs for 24 hrs and from **(D)** spleen and liver of BALB/c mice 15 days, 30days, 45 days and 60 days post-infection. **(E)** Mature miR146a-5p RNA levels were also measured from peritoneal lavages, spleen, liver and BMDMs isolated from *L*. *donovani* infected BALB/c mice euthanatized three months post-infection. PBS injected animals served as a control group and each group is representative of n = 3 animals. All the miR146a expressions were normalized against U6 snRNA. BMDMs, transfected with either non-coding RNA (anti-NC) or mirVANA miR146a-5p inhibitor oligo RNA (anti-146a), were challenged24 hrs post-transfection, with CFSE stained or unstained parasites. **(F, G)** 4 hrs post infection, percentage of CFSE-labeled parasite uptake in Cd11b^+^BMDMs was determined using flow cytometry and represented as histogram. Uptake of pHrodo green zymosan particle by uninfected BMDMs, was used as positive control. **(H)** 24 hrs post infection, survival rate of unstained parasites was measured by geimsa staining and represented as number of amastigotes per 100 BMDMs. **(I)** Western-blotting of phagocytic marker proteins DC-SIGN and Siglec-E from uninfected, anti-NC and anti-146a treated BMDMs lysates was performed, where β-actin was used as loading control. Each experiment has been performed in triplicates. Statistical significance (**P<0.01, ***P<0.001) was calculated using one-way ANOVA and data are represented as mean± SD (Graph pad prism 5.0).

We first performed real time PCR (RT-PCR)-based differential expression analysis of a group of immune-regulatory miRNAs (mmu-miR181a-5p, mmu-miR146a-5p, miR26a-5p and miR125a-5p) during *L*. *donovani* infection. We observed enrichment of miR146a-5p, miR181a-5p and miR125a-5p which are involved in M2 polarization **[21,29,30]** with concomitant depletion of M1 regulating miR26a-5p **[31]** in infected BMDMs (Fig 1A). From this screening, miR146a-5p was selected for further mechanistic evaluation of its involvement in infection-induced M2 polarization.

Next, BMDMs were infected with virulent *L*. *donovani* promastigotes at increasing multiplicity of infection ratios (MOIs) ranging from 1:0.5 to 1:20 (macrophages: parasites) and times of infection (from 4 hrs to 48 hrs). RT-PCR experiment based analysis of mature miR146a-5p titer suggested that miR146a-5p level enhanced in positive correlation with doses and times of parasite infections (Fig 1B and 1C). To correlate *in vitro* findings with *in vivo* infection, miR146a-5p expressions were also evaluated in spleen and liver of 15, 30, 45 and 60 days infected animals, which showed a successive enrichment pattern of the miRNA with time of infection (Fig 1D). Finally, miR146a-5p expression was validated in spleen, liver, peritoneal lavage and BMDMs isolated from three months (90 days) infected BALB/c mice. RT-PCR data confirmed that besides spleen and liver, miR146a-5p expression got enriched in other infected animal organs as well compared to PBS injected healthy animals (Fig 1E). These findings cumulatively indicated possible involvement of miR146a-5p during *L*. *donovani* infection.

### miR146a-5p is crucial for parasite phagocytosis and survival inside BMDMs

In order to understand the significance of miR146a-5p for parasite entry and survival inside BMDMs, macrophages were transfected with anti-NC and anti- miR146a-5p inhibitor oligos. Post-transfection, one set of BMDMs were infected with CFSE-labeled *L*. *donovani* and 4 hrs post infection, BMDMs were labeled with anti Cd11b-Alexa Flour 700 (Cd11b-AF700) antibodies. Percentage of CFSE signal reflects the amount of parasite phagocytized. Flow cytometry analysis suggested that infected Cd11b^+^ cells (INF) exhibited complete phagocytosis (100%) of CFSE-labeled parasites and anti-NC RNA transfected Cd11b^+^cells displayed 88% uptake of CFSE-labeled parasites. Surprisingly, miR146a-5p inhibitor-transfected cells displayed only 16% uptake of CFSE-labeled parasites, which is 5.5 times less than anti-NC treated cells and 6.25 times less than infection only-Cd11b^+^ cells (Fig 1F and 1G). However phagocytic capacity of healthy macrophages were assessed uptake of pHrodo green zymosan particles during the assay, which showed significant uptake of the particles. Moreover expressions of C-type lectin receptor DC-SIGN and I-type lectin receptor Siglec E were evaluated upon inhibition of miR146a-5p. Immuno-blotting data showed that infection persuaded upregulation of DC-SIGN and Siglec E, which subsequently got abrogated upon inhibition of miR146a-5p (Fig 1I). These data cumulatively suggested that miR146a-5p might play a vital role in parasite phagocytosis in BMDMs.

Similarly another set of BMDMs were infected with unstained parasites for 24 hrs. Geimsa staining based parasite burden calculation corroborated with the flow cytometry data. Parasite burden of miR146a-5p inhibitor treated BMDMs lowered significantly compared to infection only and anti-NC-treated groups (Fig 1H). These findings suggested plausible involvement of miR146a-5p dependent survival of parasites inside macrophages.

### miR146a-5p skews infected macrophage profile from M1 to M2 type during *L*. *donovani* infection

**Fig 2:**
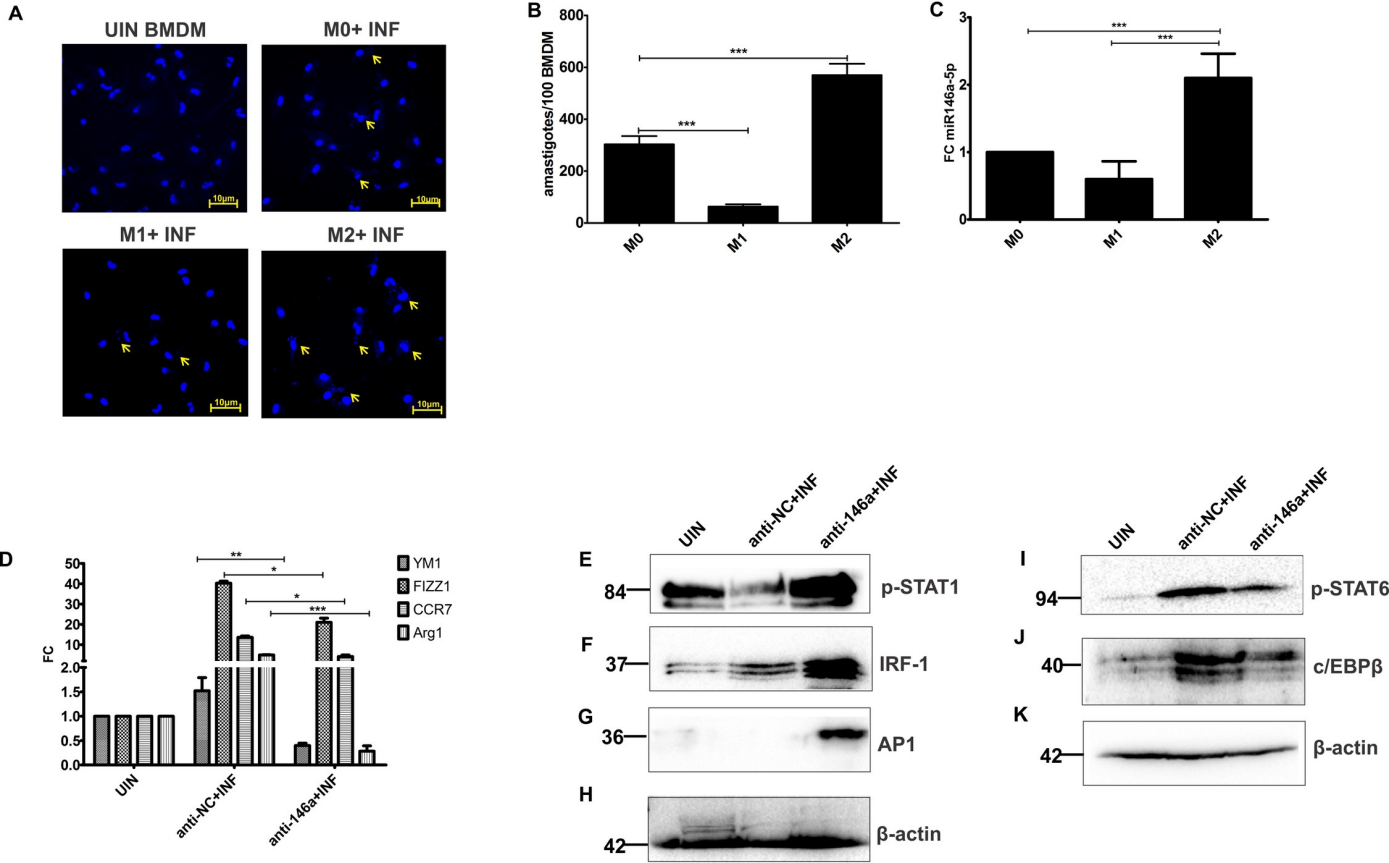
miR146a-5p is crucial for inducing M2 polarization and depleting M1 polarization markers in *L*. *donovani* infected BMDMs. Naive (M0), M1 and M2 polarized BMDMs were challenged with *L*. *donovani* at 1:10 MOI for 24 hrs, and infectivity index was determined by **(A)** confocal micrograph of Hoescht stained BMDMs (scale bar- 10 μm) followed by **(B)** geimsa staining based calculation of amastigote burden per 100 BMDMs in 100X oil immersion microscope. **(C)** Expression of miR146a-5p was determined in M0, M1 and M2 polarized BMDMs by RT-PCR. Data represented as mean ± SD. **(D)** Expression of M2 polarization markers were analyzed in *L*. *donovani* infected BMDMs previously transfected with either anti-NC or anti-146a oligos. Expression of transcription factors essential for directing either M1 **(E-H)** or M2 **(I-K)** polarization of macrophages, were determined in anti-NC or anti-146a transfected and *L*. *donovani* infected BMDMs by western blotting, where β-actin served as loading control. Statistical significance (*P<0.05, **P<0.01,***P<0.001) was calculated using one-way ANOVA and data are represented as mean± SD (Graph pad prism 5.0).

Next, we explored the infectivity index and miR146a-5p expression profile in naïve BMDMs (M0), M1 BMDMs polarized by LPS and recombinant-IFN γ, and M2 macrophages polarized by recombinant-IL4 treatment. Both confocal micrography and geimsa staining showed highest infection burden in M2 polarized BMDMs (Fig 2A and 2B).

Additionally, heightened expression of miR146a-5p was observed in M2 polarized BMDMs compared to the naïve (M0) and M1 polarized ones (Fig 2C).These finding indicated a positive relationship between miR146a-5p and M2 plasticity of macrophages.

Next differential expression profiling of M2 polarization markers from anti-NC or anti-miR146a-5p-treated and *L*. *donovani*- infected BMDMs was performed. RT-PCR data suggested a constant decrease of M2 marker genes (YM1, FIZZ1, CCR7 and Arg1) after inhibition of miR146a-5p, which were otherwise enriched in anti-NC treated BMDMs following infection (Fig 2D).

Besides surface markers, expression profiling of some transcription factors has been undertaken to elucidate global regulation of miR146a-5p upon M2 polarization. Western blot showed upregulation of p-STAT1, IRF-1 and AP1 in anti-146a-transfected BMDMs compare to anti-NC treated ones (Fig 2E–2H). On the contrary, pSTAT6 and c/EBPβ levels decreased significantly upon inhibition of miR146a-5p (Fig 2I–2K), subsidizing the role of miR146a-5p in infection induced M2 polarization of BMDMs.

### Expression of TRAF6, IRAK1 and *i*NOS got augmented upon inhibition of miR146a-5p during *L*. *donovani* infection

**Fig 3:**
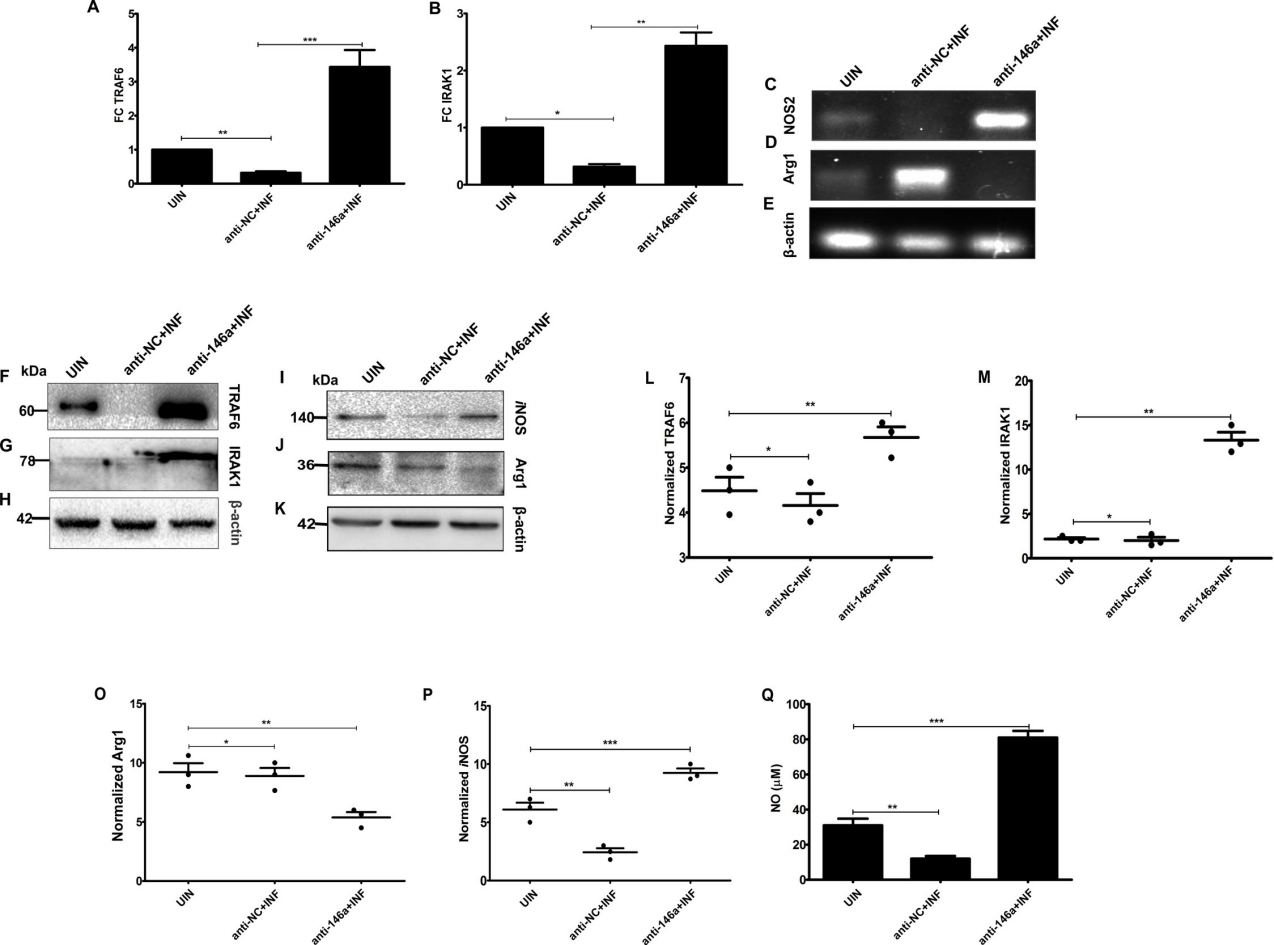
Inhibition of miR146-5p induces enrichment of TRAF6, IRAK1 and *i*NOS expression and Arg1 depletion in *L*. *donovani* infected BMDMs. **. (A)** Expressions of miR146a target genes, namelyTRAF6 **(A, F, L)** and IRAK1 **(B, G, M)** were evaluated by RT-PCR and immune-blotting. **(C, D, E)** Semi-quantitative PCR of NOS2 and Arginase1 (Arg1) genes was performed followed by electrophoresis of the PCR products in 1% agarose gel. Protein level expressions of *i*NOS **(I, P)** and Arg1 **(J, O)** were determined by western blotting and **(Q)** nitric oxide titer in culture supernatant was measured by Griess assay. Mouse β actin served as reference gene for PCR experiments. Each experiment has been performed in triplicates. Statistical significance (*P<0.05, **P<0.01,***P<0.001) was calculated using one-way ANOVA. Data represented as mean± SD.

Previous reports suggested that miR146a-5p targets TRAF6 and IRAK1 to negatively regulate NF-κB pathway and *i*NOS generation by macrophages **[32].** Hence, we investigated both RNA and protein expression patterns of TRAF6, IRAK1, *i*NOS and Arg1 genes in anti-NC or anti-146a treated and infected BMDMs. Both RT-PCR and western blot data showed significant enrichment of TRAF6 (Fig 3A, 3F and 3L) and IRAK1 (Fig 3B, 3G and 3M) in anti-miR146a transfected BMDMs upon parasite challenge, compared to anti-NC transfected BMDMs.

M1 polarized macrophages are characterized by low Arginase 1 (Arg 1), high induced nitric oxide synthase (*i*NOS) and enhanced nitric oxide levels. Our study revealed that expression of *NOS2* gene (Fig 3C), *i*NOS protein (Fig 3I and 3P) and NO (Fig 3Q) were significantly higher with subsequent downregulation in Arg1 expression level (Fig 3D, 3J and 3O) in anti-miR146a treated BMDMs, 24 hrs post-infection compared to the anti-NC treated ones. These observations clearly suggested that miR146a-5p aids in negative regulation of M1 polarization by down regulating TRAF6, IRAK1 and *i*NOS expressions during *L*. *donovani* infection.

### *L*. *donovani* infection induces anti-inflammatory M2 cytokines in a miR146a-5p dependent manner

**Fig 4:**
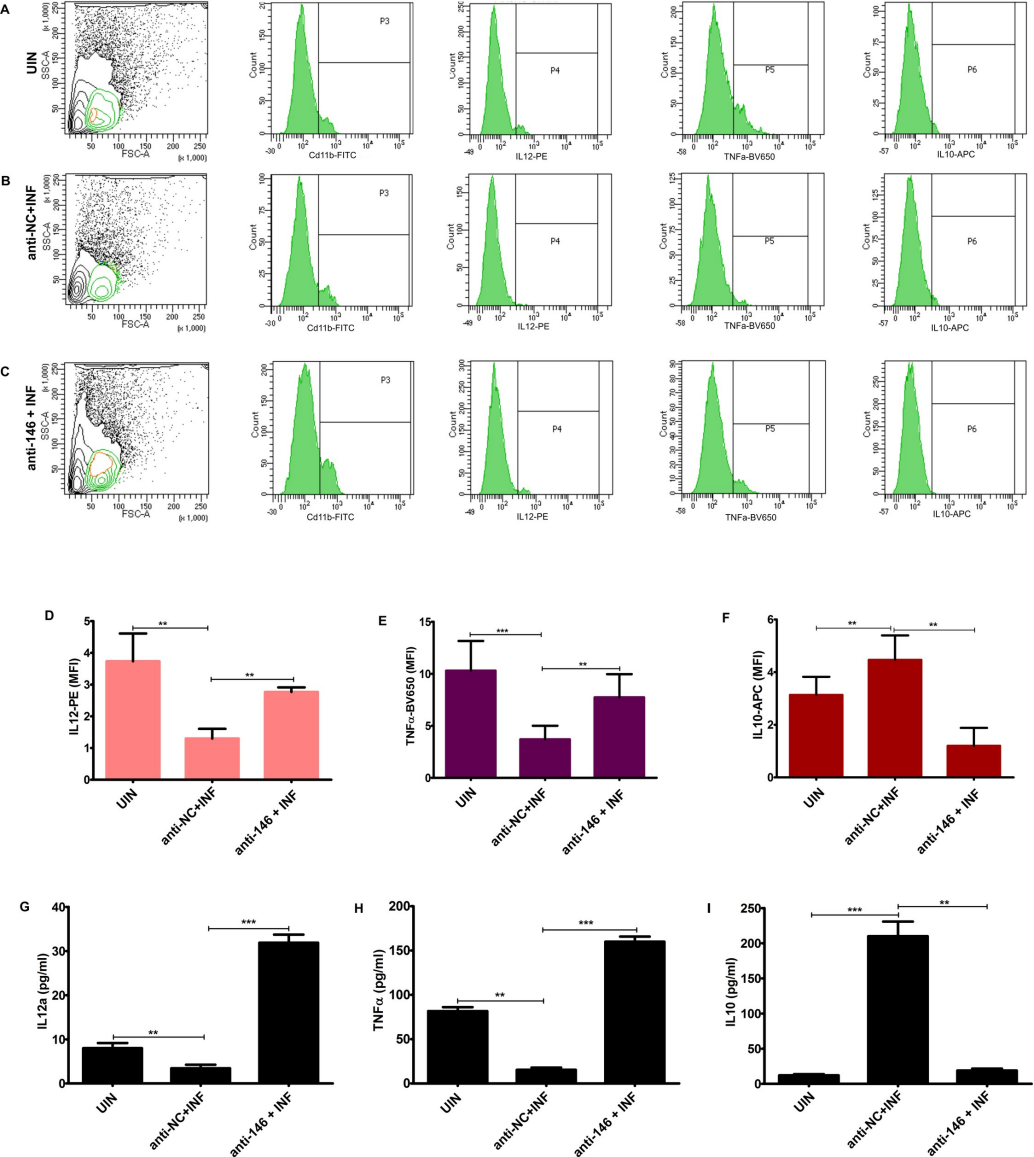
miR146a-5p is essential for *L*. *donovani* infection-mediated enrichment of anti-inflammatory M2 cytokines. BMDMs, transfected with anti-NC RNA and anti-miR146a-5p inhibitor oligos, were infected with *L*. *donovani* parasites for 24 hrs. **(A-C)** Cytokine expression profile was recorded from Cd11b^+^ populations of BMDMs stained with IL10-APC, IL12- PE and TNFα-BV650 antibodies. **(D-F)**Cytokine expressions were recorded using auto polygon gating strategy in BD FACSDiva (from compensated unstained control) and represented using histogram plots. Mean fluorescence intensity (MFI) of each cytokine was further represented using bar diagrams. **(I-K)** Cytokines profiling, was performed by sandwich ELISA, using culture supernatant isolated from macrophages. Statistical significance (*P<0.05, **P<0.01,***P<0.001)was calculated using one-way ANOVA. Data represented as mean± SD.

Polarity of macrophages upon pathogenic challenge largely depends upon its cytokine profile. Upon entry into the macrophages, *L*. *donovani* parasite enhances anti-inflammatory cytokines synthesis which aids in survival and proliferation of the amastigotes **[15].** Therefore, we explored effect of miR146a-5p inhibition upon cytokine profile of infected BMDMs by multi-color flow cytometry and sandwich ELISA. FACS analysis showed that parasites were unable to increase IL10 levels in anti-miR146a-5p transfected BMDMs compared to anti-NC transfected ones (Fig 4A–4C and 4F). On the contrary, mean fluorescence intensity of IL12 and TNFα increased significantly upon inhibition of miR-146a-5p (Fig 4A–4E). These observations were further supported by determination of secreted cytokine titers in culture supernatants by ELISA, which also showed exhaustion of IL10 titer and enrichment of IL12 and TNFα in miR-146a inhibited BMDMs upon parasite challenge (Fig 4G–4I). Cumulatively, we found that miR146a-5p is crucial for enrichment of M2 cytokines during infection.

### Nuclear translocation of phosphorylated p65 subunit got stimulated upon miR146a inhibition

**Fig 5:**
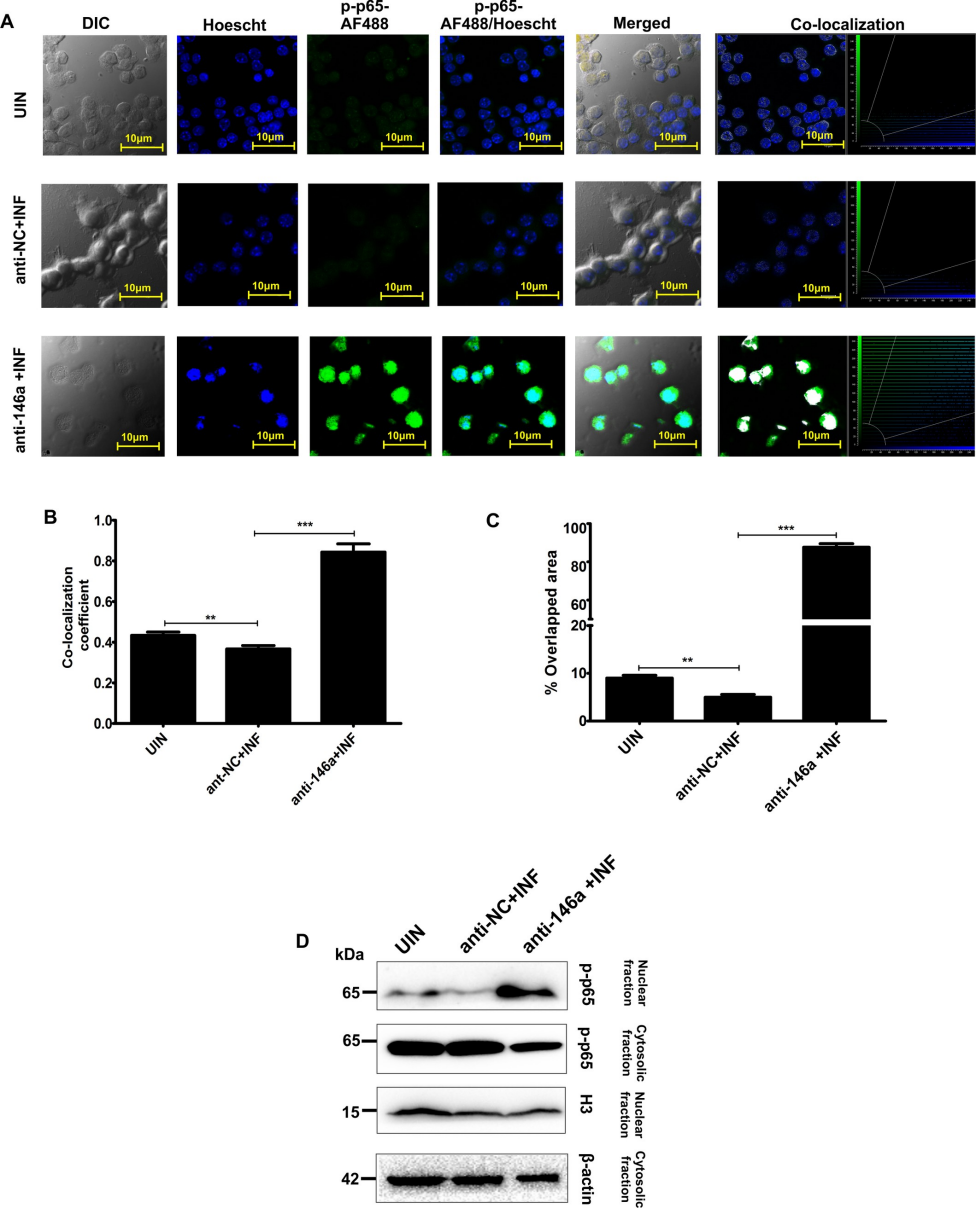
Nuclear translocation of phosphorylated p65 subunit got suppressed during *L*. *donovani* in miR146a-5p dependent manner. **(A)** Nuclear translocation of phospho-p65 (ser536) in anti-NC and anti-146a inhibitor transfected BMDMs was evaluated by confocal microscopy of infected BMDMs. **(B, C)** Co-localization coefficients of p-p65 alexa Fluor 700 (AF700) signals with Hoescht signals were determined while capturing the images at 63X oil immersion objective with 2.0X optical zoom factor (Scale bar: 10 μm). Post hoc analysis was performed using Leica Application Suit X (LAS-X software) and co-localization coefficients with percent overlap of the signals were plotted for each condition. **(D)** Nuclear and cytosol fractions of the BMDMs were isolated and expression of p-p65 was analyzed by western blotting. Histone H3 and β-actin are used as loading controls for nuclear and cytosolic fractions respectively. Statistical significance (**P<0.01,***P<0.001)was calculated using one-way ANOVA. Data represented as mean± SD.

miR146a-5p is a major negative feedback regulator of NF-κB pathway. Therefore we evaluated the effect of parasite infection upon nuclear translocation of p-p65 by both confocal microscopy and western blotting. Co-localization co-efficient values and percent overlap of pp65 signals with nuclear Hoescht signals suggested positive nuclear accumulation of phospho-p65 subunit (ser536) in miR-146a-5p inhibited and *L*. *donovani* infected BMDMs compared to anti-NC transfected cells (Fig 5A–5C). Immuno-blotting of p-p65 subunit in nuclear and cytosol fractions of anti-NC or anti-146a transfected BMDMs also substantiated that nuclear retention of p-p65 subunit enhanced in infected BMDMs upon inhibition of miR146a-5p (Fig 5D). Interestingly, parasites were unable to suppress phosphorylation as well as nuclear translocation of p65 subunit upon deletion of miR146a-5p which indicated the microRNA as a major nexus for *L*. *donovani* infection-based suppression of NF-κB pathways.

### BRD4/p300 dependent super enhancers drive expression of macrophage miR146a-5p during *L*. *donovani* infection

**Fig 6:**
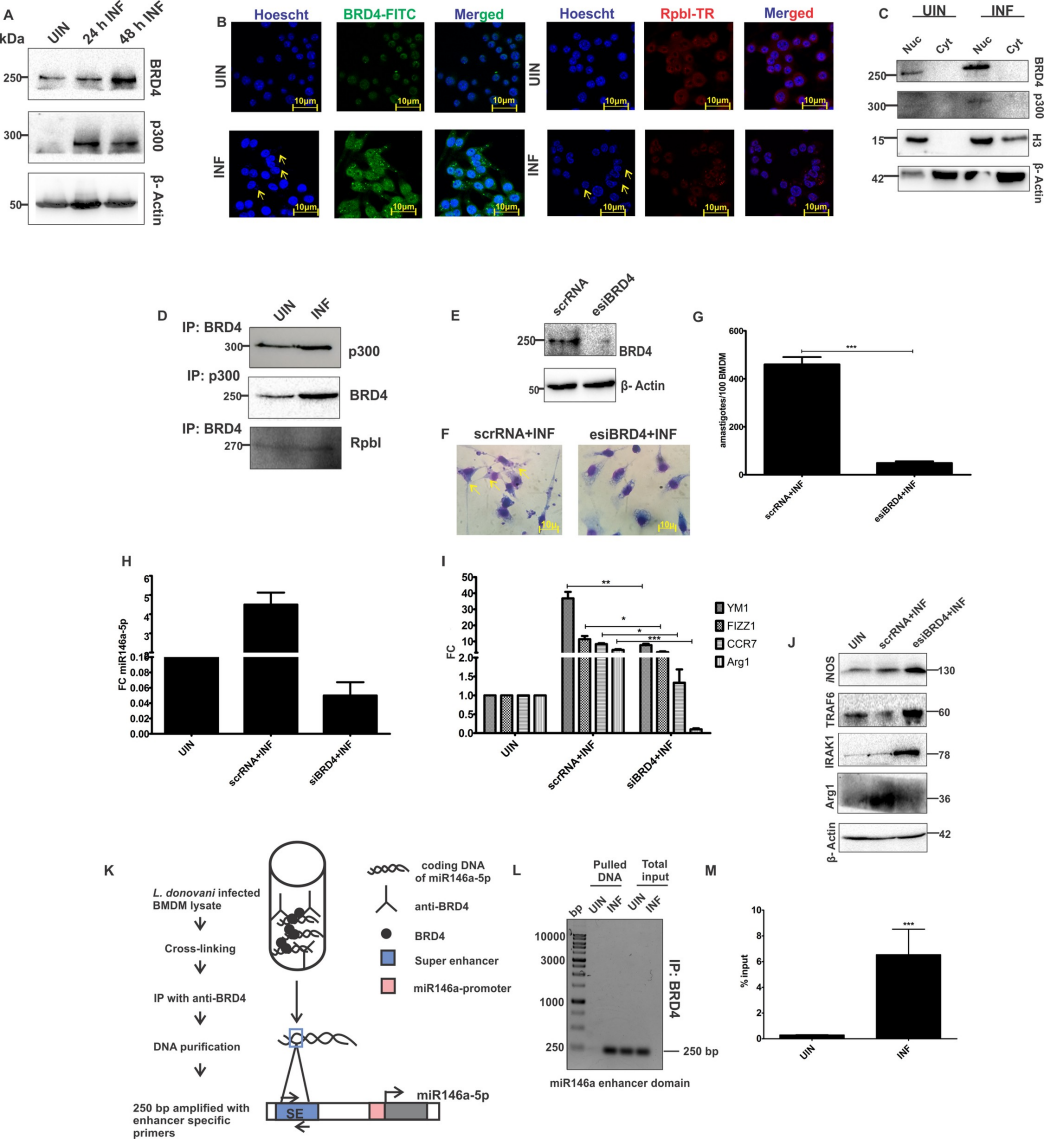
BRD4 mediated super enhancer complex ensures prolonged enrichment of miR146a-5p and M2 polarization profile of *L*. *donovani* infected BMDMs. **(A)** Western blotting of major super enhancer proteins like BRD4 and p300 in *L*. *donovani* infected BMDMs. **(B)** Nuclear localization of BRD4 and RNA polymerase subunit I (RpbI) were determined by confocal micrographs of uninfected and infected BMDMs. Representative images were obtained at 63X oil immersion objective (scale bar-10μm) with 2.0 zoom factor and processed using LAS-X software.**(C)** Nuclear and cytosolic fractions of infected BMDMs were subjected to western blotting for BRD4 and p300. Nuclear translocated proteins were normalized with Histone H3. **(D)** Western blot of p300 and RpbI, immune-precipitated by anti-BRD4 antibody and western blot of BRD4, immuno-precipitated by anti-p300 antibody using co-immuno precipitation assay. **(E)** BMDM lysates, transfected with scrambled RNA (scrRNA) or enhanced siRNA pool for BRD4 (esiBRD4) were subjected to western blotting for evaluating BRD4 silencing efficiency 24 hrs post-transfection. **(F, G)** BRD4 silenced BMDMs were infected with *L*. *donovani* parasites and 24 hrs post-infection infectivity indexes were determined by geimsa staining and expressed as number of amastigotes per 100 BMDMs. Images of stained field was procured in 100X oil immersion objective (scale bar- 10μm). **(H, I)** Expression of mature miR146a-5p and M2 polarization marker genes were analyzed by RT-PCR in scrRNA and esiBRD4 treated and infected macrophages**. (J)** Western blot of miR146a-5p target genes (TRAF6, IRAK1), *i*NOS and Arg1 was done from lysates of scrRNA and esiBRD4 treated and infected macrophages**. (K)** Schematic diagram of chemical cross-linking based chromatin immune-precipitation of super enhancer region of miR146a-5p gene using anti-BRD4 antibody. **(L)** Semi-quantitative PCR of enhancer DNA, amplified from total input and purified DNA pool precipitated by anti-BRD4 antibody, followed by gel electrophoresis in 1.5% agarose gel. **(M)** RT-PCR based quantification of BRD4 protein enrichment at miR146a-5p enhancer region, using DNA purified from ChIP experiments. Percent input represents normalized amount of enhancer DNA enriched against total input. Statistical significance (*P<0.05, **P<0.01, ***P<0.001) was calculated using one-way ANOVA. Data represented as mean± SD.

Our next question was how the infection mediates sustained and prolonged expression of mature miR146a-5p RNA. Super enhancer (SE) elements are outstanding cis-acting elements, which transcriptionally control sustained RNA expression under different stimulations. BET bromo domain protein BRD4, p300 and RNA pol II accumulate at these SE complexes for induction of enhanced expression of genes. Bioinformatics analysis of protein-protein interaction using STRING v.11.0 showed a plausible interaction amongst BRD4, p300, RNA pol II and histone H3 (node interaction cut off <0.05, S1 Fig). We therefore experimentally validated formation of SE complexes during *L*. *donovani* infection at miR146a-5p gene loci of mouse BMDMs. Immunoblotting of whole cell lysate and nuclear fractions suggested significant upregulation and nuclear translocation of BRD4 and p300 during *L*. *donovani* infection (Fig 6A and 6C). Besides that, confocal microscopy based validation of nuclear retention of BRD4 as well as RpbI also substantiated the western blot data (Fig 6B). Moreover immune-precipitation studies showed a strong interaction between these three proteins (Fig 6D). Immune reactive bands of p300 and RpbI developed upon pull-down with anti-BRD4 antibodies and BRD4 protein bands developed upon pulling down with anti-p300 antibodies from infected BMDM lysate (Fig 6D). These findings clearly denoted that BRD4, p300 and RNA pol II are translocating in infected BMDM nuclei and having strong interactions amongst themselves.

BRD4 expression was successfully silenced with enhanced siRNA pool (esiRNA) (Fig 6E). Geimsa staining followed by amastigote count showed compromised parasite burden of esiBRD4 transfected BMDMs compared to scrRNA transfected ones (Fig 6F and 6G). Interestingly, parasite infection was unable to induce miR146a-5p expression in BMDMs after silencing BRD4, which indicated involvement of BRD4 in transcription of miR146a-5p (Fig 6H). Upon infection, expression of M2 polarization markers got down regulated in esiBRD4 transfected BMDMs compared to scrRNA transfected ones (Fig 6I). TRAF6 and IRAK1 as well as *i*NOS expression increase with concomitant exhaustion of Arg1 expression in esiBRD4 transfected BMDMs (Fig 6J). Finally, to establish the SE formation, we pulled down chromatin region bound by BRD4 protein by chemically cross-linked chromatin immune-precipitation (X-ChIP) (Fig 6K). DNA population pulled down by BRD4 antibodies from uninfected and infected BMDMs were subjected to semi-quantitative PCR and RT-PCR. 250 bp length amplicon of BRD4 protein bound mir146a-5p enhancer region got enriched in infected BMDMs (Fig 6L).As per RT-PCR data, the amount of percent input of the BRD4 bound DNA from infected BMDMs was significantly higher compared to uninfected ones (Fig 6M). Taken together, these data affirmed SE complex driven transcription of miR146a-5p during *L*. *donovani* infection.

### miR146a-5p is indispensable for *in vivo* infection

**Fig 7:**
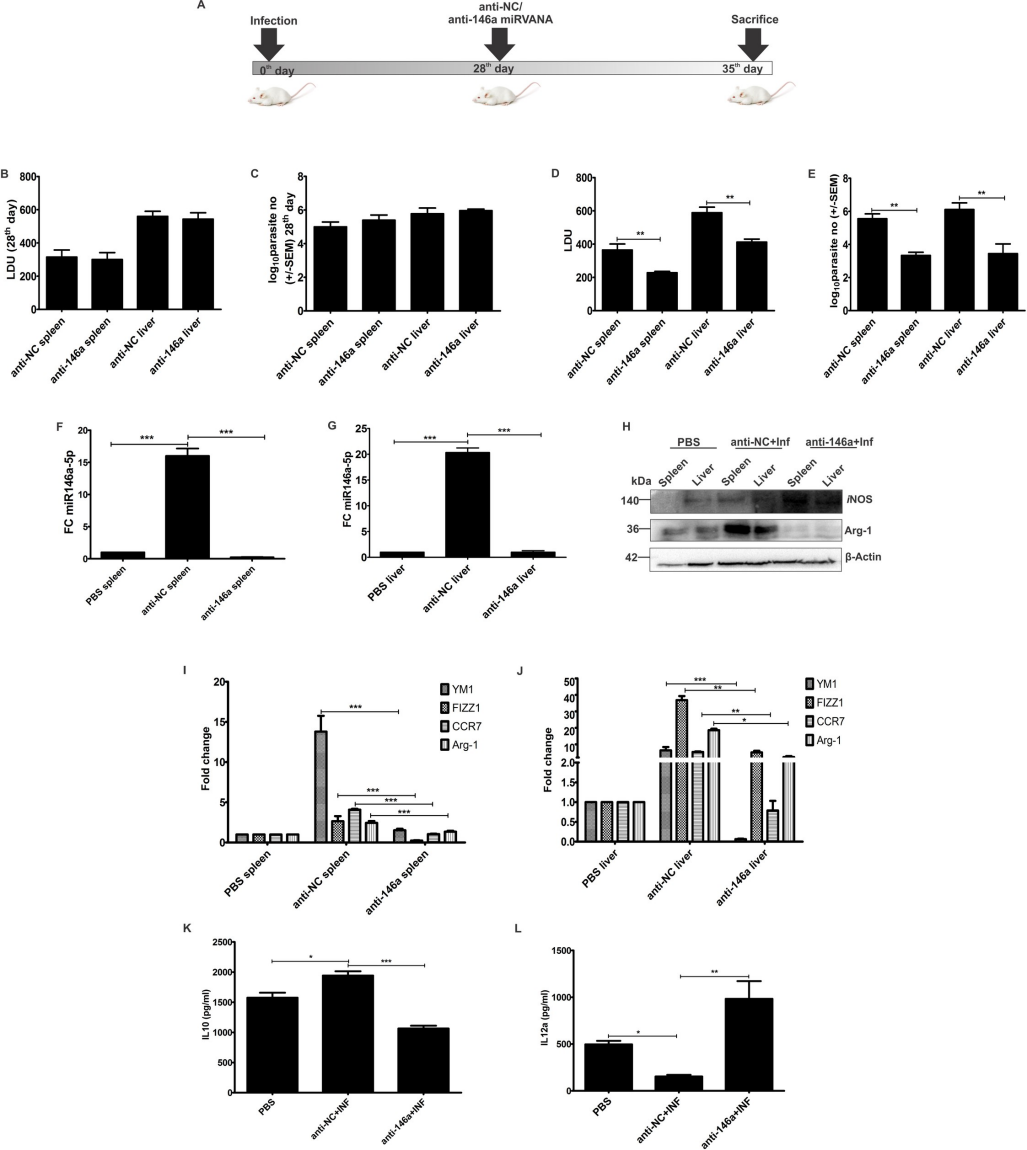
Parasite burden and M2 polarity markers got abrogated in miR146a inhibited BALB/c mice organs. **(A)** 4–6 weeks old BALB/c mice were infected with 2X10^7^ *L*. *donovani* suspended in 1.8% glucose- phosphate buffered saline solution. Before administration of anti-NC and ant-146a oligos, 28-days post-infection, infection burden of animals from each group were checked by **(B)** Leishman Donovan unit (LDU) calculation of geimsa stained stamped smears of spleen and liver and **(C)** limiting dilution assay (LDA) of 1mg/ ml (w/v) minced organ suspensions. 28 days post-infection, 0.025 mg/g body weight anti-NC oligos and mirVANA anti-146a inhibitor were injected in two separate groups of mice (n = 3 animals/ group) via tail vein route. PBS injected animals were used as healthy control group for the study.7 days post PBS or inhibitor treatment animals were euthanized. Parasite burden of spleen and liver was calculated by LDU **(D)** and LDA**(E)**, where LDA is displayed as Log10 scale graph and LDU parasite count is expressed as parasites count per organ.**(F, G)** miR146a-5p titer in spleen and liver of PBS, anti-NC and anti-146a injected animals was determined by RT-PCR where U6 snRNA was used as reference gene.**(H)** Western blotting of spleen and liver lysate procured from PBS, anti-NC and anti-146a injected animals was performed for *i*NOS, Arg1 expression evaluation and β- Actin was used for normalization. (**I, J**) Expression profile of M2 polarization markers were analyzed in organ RNA and normalized using β- Actin.(**K, L**) Splenocytes of three experimental groups were cultured overnight in presence of 10 μg/ml leishmania antigen (LAg) stimulation, followed by ELISA-based evaluation of secreted IL10 and IL12 cytokines titer in splenic culture supernatants. Each experimental data set is representative of n = 3 animals and error bars as mean ± SD. Statistical significance was analyzed using one-way ANOVA followed by Dunnett’s multiple comparison test (*P<0.05, **P<0.01, ***P<0.001).

Previously infection time kinetics suggested a succeeding upregulation of miR146a-5p in BALB/c mice till three months post-infection (Fig 1D and 1E). In order to understand significance of the miRNA for infection persistence, we have for the first time established *in vivo* miR146a-5p neutralized animal model for VL, by injecting miRVANA antagomirs anti-146a RNA via intravenous route in 28 days infected animals (Fig 7A). Before injecting inhibitor oligos, infection burden in two animal groups were analyzed by Leishman donovan unit (LDU) counting and Limiting dilution assay (LDA) of spleen and liver. In both the organs parasite successfully established synchronized infection (Fig 7B and 7C). 28-days post infection, anti-NC and anti-146a oligos were injected in respective animal groups. PBS injected age-matched healthy animals were kept as controls.

7 days post-injection of PBS, anti-NC and anti-146a oligos, animals were sacrificed followed by determination of organ parasite burden. LDU counting and LDA showed decreased organ amastigote burden in the miR146a-5p neutralized animal group compared to anti-NC injected animals (Fig 7D and 7E). In order to ensure the efficacy of miRVANA oligos, miR146a-5p expression was evaluated in anti-NC and anti-146a injected animal spleen and liver. RT-PCR data suggested that miR146a-5p pool got successfully neutralized upon injection of inhibitor oligos (Fig 7F and 7G).

Subsequently by immune-blotting we observed that expression of Arg-1 decreased and that of *i*NOS increased in organ lysates of miR146a-5pneutralized group compared to anti-NC treated group (Fig 7H). Differential expression analysis of M2 polarization markers in the organ (spleen and liver) RNA indicated that, expression of M2 markers decreased significantly in spleen and liver of miR146a-5p neutralized animals compared to the anti-NC treated groups (Fig 7I and 7J). Moreover, cytokine-ELISA of LAg stimulated splenocytes supernatant from infected and PBS/anti-NC/anti-146a treated animals, showed downregulation of IL10 titer (Fig 7K) and upregulation of IL12 titer upon neutralization of miR146a-5p compared to the anti-NC treated groups (Fig 7L). Thus, we recognized a role of miR146a-5p for *in vivo* infection persistence and establishment of immune-suppression in host.

## Discussion

Macrophages are the major antigen presenting cells of the innate immune system which provide first line of defense by phagocytosis of breaching pathogens **[33]**. The immunological status of the macrophages switches between immune reactive M1 and immune suppressive M2 types, depending upon the cues present in the microenvironment **[7]**. *L*. *donovani* proliferates within the host macrophages by triggering immune suppressive conditions **[34]**. Thus host susceptibility or resistance against *L*. *donovani* infection largely depends upon the plasticity of the infected macrophages. Previous studies have shown that *L*. *donovani* infection triggers macrophage mammalian target of rapamycin (mTOR) pathway, as well as peroxisome proliferator-activated receptor gamma (PPAR-γ) and CD163 expression, thereby facilitating the enrichment of Arg1-mediated polyamine synthesis and M2 polarization of macrophages **[35–37]**. These studies underlined that parasites skew macrophages towards M2 type. Small RNA profiling of *L*. *donovani* and *L*. *major* infected patient PBMCs, THP-1, and dendritic cells showed virulent parasites mediated differential enrichment of immune regulatory miRNAs **[17,38]**. However, the role of these miRNAs in regulating macrophage polarization during *L*. *donovani* infection is still poorly understood. In our current study, differential expression profiling revealed infection-dependent enrichment of miR146a-5p, miR181a-5p andmiR125a-5p and depletion of miR26a-5p which paralleled with the hypothesis that *L*. *donovani* infection affects upon the polarization-regulating miRNA landscape of macrophage. miR26a-5p promotes M1 polarization via targeting Kruppel like factor 4 (KLF4) and c/EBPβ **[31]**. Additionally, miR181-5p and miR125a-5p were previously shown to have substantial role in inducing M2 polarization via targeting c/EBPα-KLF6 axis and KLF13 respectively **[29,30]**.However, amongst all the M2 regulating miRNAs, miR146a-5p showed maximum enrichment during *L*. *donovani* infection. Recently, by chemical circuit-based and systems biology-based approaches, Nimsarkar *et al*. discovered that *L*. *major* exports miR146a-5p like elements in infected THP-1 cells, which in turn targets SMAD7, a mediator of TGFβ signaling network **[22]**. Such findings instigated us to explore mechanistic prominence of miR146a-5p in targeting another major immune-regulatory arm like TLR4 signaling mediators TRAF6 and IRAK1 and its effect upon macrophage polarization during *L*. *donovani* infection. Recent reports highlighted transcription of miRNA is pedantically regulated by some extraordinary epigenetic elements called super enhancers (SEs) **[24]**. We deciphered thatBRD4-p300-RNA pol II mediated SE complex regulates persistent transcription of miR146a-5p in *L*. *donovani* infected macrophages where BRD4 plays an indispensable role.

Available experimental reports suggest that miR146a-5p got enriched in *Mycobacterium tuberculosis* infected THP-1 cell line and organs of BALB/c mice **[39]**.Currently we also found that miR146a-5p exhibited an exponential enrichment pattern in *L*. *donovani* infected bone marrow derived macrophages (BMDMs) which positively correlated with dose and time of infection. Additionally, we obtained miR146a-5p enrichment in the infection foci of BALB/c mice like spleen, liver, BMDMs and peritoneal lavages. These cumulatively postulated that *L*. *donovani* infection is possibly triggering a long and persistent expression of miR146a-5p.

When *L*. *donovani* infection onsets in host, spleen, liver and peritoneal regions become the foremost local infection foci. The infection spreads into remote organs like bone marrow in later stage of long-term chronic infection **[40]**. Therefore, the differential enrichment pattern in infected animal organs suggested that miR146a-5p exhibits maximum enrichment in chief infection foci compared to distal organs three months post infection. Previously it was reported that in *in vitro Mycobacterium bovis* infected RAW 264.7 cell line, miR146a expression enriched whereas in tuberculosis patient alveolar lavage, miR146a-5p expression was unaltered **[41]**. This advocated that in primary cells, miRNA expression alteration is not always directly linked with infection **[42]**. With this notion in hand, we temporarily blocked the mature transcripts of miR146a-5p by transfecting miRVANA miR146-5p inhibitors in BMDMs, to investigate whether parasite persistence is interrelated with miR146a-5pexpression of BMDMs.miR146a has previously been reported as a critical regulator for phagocytosis of pathogens like Dengue virus, Enterovirus 71, *Listeria monocytogenes*, *M*. *tuberculosis* etc.**[39,43]**. Phagocytosis rate of *L*. *donovani* decreased dramatically in BMDMs transfected with miR146a-5p inhibitor RNA, which is suggestive of an association of miR146a-5p in the entry as well as persistence of *L*. *donovani* within BMDMs. Furthermore, infection induced expression of dendritic cell-specific ICAM-3-grabbing nonintegrin (DC-SIGN) and Siglec E got abrogated upon inhibition of miR146a-5p. Literature suggest the interfering role of miR155 upon c-type lectin receptor DC-SIGN, aiding in suppression of pathogen binding with antigen presenting cells **[44]**. C-type lectin receptor DC-SIGN has substantial role in *Leishmania sp*. promastigote attachment and entry in macrophages and dendritic cells **[45]**. Similarly, I-type lectin receptor Siglec E reportedly interacts with sialic acid of promastigotes surface, aiding in entry of parasites [46]. Our finding evidenced that, by simultaneous depletion of DC-SIGN and Siglec E, miR146a-5p plausibly interferes with parasite phagocytosis by BMDMs.

Conflicting evidences exist regarding the role of miR146a in macrophage polarization. In TNFα-stimulated M1 type PBMCs of rheumatoid arthritis patients, expression of miR146a was found to be increased **[47]**. But recent findings highlighted the role of miR146a in promoting M2 polarization by inducing PPAR-γ and targeting Notch1 as well as inhibin A in RAW 264.7cells (INHA) **[21,48,49]**. Moreover, in *M*. *tuberculosis* infected THP-1 macrophages, miR146a was found to suppress nitric oxide biosynthesis by negatively regulating NF-κB signaling **[50]**. Herein, we also found miR146a-5p expression enriched significantly in recombinant mouse IL4-stimulated M2 BMDMs compared to naïve and LPS/ IFNγ-stimulated M1 macrophages. RT-PCR based expression profiling of M2 polarization markers and transcription factors in anti-NC treated and *L*. *donovani* infected BMDMs, suggested that infection induces M2 polarization of macrophages. Concomitantly, infection dependent enrichment of M2 markers got abrogated along with upregulation of M1-stimulating transcription factors upon inhibition of miR146a indicated a credible link between miR146a-5p enrichment and M2 polarization during *L*. *donovani* infection.

miR146a-5p is known to negatively regulate host NF-κB mediated activation of *i*NOS and promote Arg1 via suppression of TRAF6 and IRAK1**[51]**. We observed that TRAF6 and IRAK1 were down regulated during *L*. *donovani* infection which got abrogated after inhibition of miR146a-5p. Nitric oxide generation and exhaustion of Arg1 expression are major indicators of successful M1 macrophage polarization **[33]**. Moreover expression of *i*NOS decreased in BMDMs infected with *M*. *tuberculosis*, as a result of miR146a-5p enrichment **[42]**. Herein we found downregulation of Arg1 and abundance of *i*NOS in miR146a-5p inhibited and infected BMDMs, indicating miR146a-5p as an important node for *i*NOS depletion and Arg1 elevation during *L*. *donovani* infection. After pathogenic infections, cytokines mainly regulate the synthesis of *i*NOS and Arg1 in macrophages which ultimately determine their plasticity. In M2 macrophages, IL10 triggers Arg1 synthesis followed by inhibition of pro-inflammatory cytokines and NO generation **[52,53]**. We have seen that, upon inhibition of miR146a-5p BMDMs exhibited lowered levels of IL10 with subsequent enrichment of pro-inflammatory cytokines like IL12 and TNFα. M1 macrophages are characterized by their capacity to produce nitric oxide and reactive oxygen species (ROS) **[54]**. M1 cytokines like IL12 is mainly engaged in inducing *i*NOS generation and TNFα in activating reactive oxygen species (ROS) pathways **[55]**. We have found enhancement of nitric oxide levels during miR146a inhibition, but the role of intracellular ROS generation and the link between TNFα mediated response and miR146a-5p during VL has not been studied and we will explore it in future. TRAF6 and IRAK1 activate NF-κB p65 subunit via phosphorylation of serine 536 residue in p65 subunit followed by nuclear translocation, where p-p65 interacts with κ response elements of DNA and stimulates synthesis of M1 cytokines and *i*NOS **[56–58]**. We established that anti-146a transfected BMDMs have higher nuclear accumulation rate of p-p65, which advocate the essentiality of miR146a for dampening p-p65nuclear translocation during infection.

For macrophage immune therapy, miRNAs inhibitors namely antagomirs (miRVANA inhibitors), are recently being used due to ease of designing, stability and mode of administration in animals **[59]**. For enhanced chemical stability these antagomirs are modified by substituting the phosphate oxygen with sulfur, adding 2′-O-methyl group to non-bridging oxygen, connecting the 2′-oxygen to the 4′-carbon for locking the bridge-locked nucleic acids (LNA), or by adding a peptide **[60]**. The modified RNA inhibitors can then easily be administered through intravenous route in animals and are highly stable in blood-circular system **[59]**. These antagomirs have previously been shown to have target-specific effects upon concerned miRNAs in cancer and other preclinical disease models**[61]**. We therefore deciphered the therapeutic utilization of miRVANA antagomirs (anti-miR146a-5p inhibitors) in VL, by intravenously administering the oligos in 28-days infected BALB/c mice. We found that *in vivo* neutralization of miR146a-5p led to significant decrease inmiR146a-5p pool as well as the parasite load of infected spleen and liver. Expressions of M2 polarization markers Arg1 protein and M2 cytokine IL10 got reduced with concurrent abundances of IL12 and *i*NOS. In early 80s and 90s multiple attempts of macrophage therapy in cancer, by adoptive transfer of immune reactive macrophages inside the core of the tumors failed and the concept got abandoned **[62]**. But understanding of critical molecular players that lead to generation of M2 macrophage during infection and cancer will allow alleviation of tumor cells **[28]** or intra-macrophagic pathogens like *L*. *donovani* by activating M1 macrophages, re-empowering the above approach.

Transcriptional regulation of miRNA shares a similar mechanism to that of mRNA. Therefore it can be assumed that epigenetic modifiers are essential part of miRNA transcription. Recent reports highlighted that super enhancers (SE) drive integrative biogenesis and Drosha-DGCR8 mediated processing of immune-regulatory pri-miRNAs (miR146a and miR155) **[24,63].** SEs are a special type of cis-acting regulatory elements, which are combination of multiple enhancer-like elements, occupied by unique highly active transcriptional regulators like BRD4, p300 histone acetyl transferase, RNA pol II and active histone markers (H3K27Ac) **[63]**. SE elements represent a high order epigenetic regulation of miRNA expression, beyond their transcription activation. By undertaking a temporal kinetics of miR146a-5p expression in *L*. *donovani* infected BMDMs, a persistent pattern of miRNA enrichment was perceived from 4 hrs to 48 hrs (Fig 1B). This instigated us to explore if any robust transcriptional regulatory complex is involved behind miR146a-5p induction during *L*. *donovani* infection.

Protein interaction network analysis by STRING database **[64]** showed an interaction networking amongst BRD4, p300, RNA pol II (RpbI) and histone H3, where BRD4 got emphasized as the hub protein. We found up regulated expression and nuclear localization of BRD4 and p300 in infected BMDMs. Besides that, BRD4 and RpbI co-localized in the nucleus and immune-precipitation assay verified the interaction amongst these three SE components during infection. BRD4 is the major structural component of SE complex and JQ1 (a synthetic inhibitor of BRD4) was previously shown to inhibit SE-mediated co-transcriptional processing of miRNAs by chemically impeding BRD4 **[63]**. Thus we performed BRD4 knock down assay and found that silencing of BRD4 significantly abrogated miR146a-5p and M2 polarization marker expressions in infected BMDMs. Additionally, expression of the miR146a-5p target genes (TRAF6 and IRAK1) and *i*NOS were amplified with concurrent decrease in the protein level of Arg1. These data indicated that *L*. *donovani* infection-driven induction of miR146a-5p is BRD4-dependent. Finally we confirmed the occupancy of BRD4 at the upstream enhancer regions of miR146a-5p gene, by chemical cross-linked chromatin immune pull down assay (X-ChIP) using anti-BRD4 antibody. As per available mouse macrophage ChIP-seq analysis, consistent co-occupancy of BRD4 and RNA pol II at miR146a enhancer, corroborated that SE element directs robust expression of miR146a gene in macrophages **[24]**. SE elements are highly fragile and easily “broken” **[65]**, still we were able to rescue nearly 1 kb fragment by controlled sonication protocol during X-ChIP, and amplified 250 bp amplicons by using specific primers flanking the enhancers of miR146a-5p gene to prove BRD4 accumulated at miR146a-5p enhancer in *L*. *donovani* infected BMDMs.

Understanding of miRNA regulation during parasite infection by high order chromatin elements like SE will add a new dimension to *Leishmania* infection biology. **Parmar *et al*.** recently established that *L*. *donovani* infection mediates macrophage polarization by broad range histone make over in polarized macrophages **[34]**. Our observations additionally highlight novel roles of super enhancers and epigenetic regulator protein BRD4 in sustained miR146a-5p expression during *L*. *donovani* infection which can be a promising juncture for transcriptional perturbation based therapeutics against VL. Furthermore establishment of miR146a as a major immune regulator of macrophage polarization will open a novel avenue for therapeutic targeting of miRNA using miRVANA miR146a-5p inhibitors and will add advancement in the field of macrophage immune therapy in VL.

## Supporting information

S1 Fig STRING database reveled interaction among BRD4, p300, RpbI and Histone H3 proteins. **(A**) Protein-protein interaction prediction among BRD4, p300, RNA pol II and Histone H3 by STRING V.11. **(B)** Classification of interacting partners of BRD4 into different groups based on the interacting protein functions.
(TIF)

S2 Fig Uncropped images of the gel and immuno-blots. (TIF)

S1 Table List of primers used for real time and semi quantitative PCR reactions. (DOCX)
