# Supplementary figures and images for: Super enhancer-mediated transcription of miR146a-5p drives M2 polarization during Leishmania donovani infection
Source: PLoS Pathog. 2021 Feb 25;17(2):e1009343. doi: 10.1371/journal.ppat.1009343 (PMC7943006; doi:10.1371/journal.ppat.1009343)

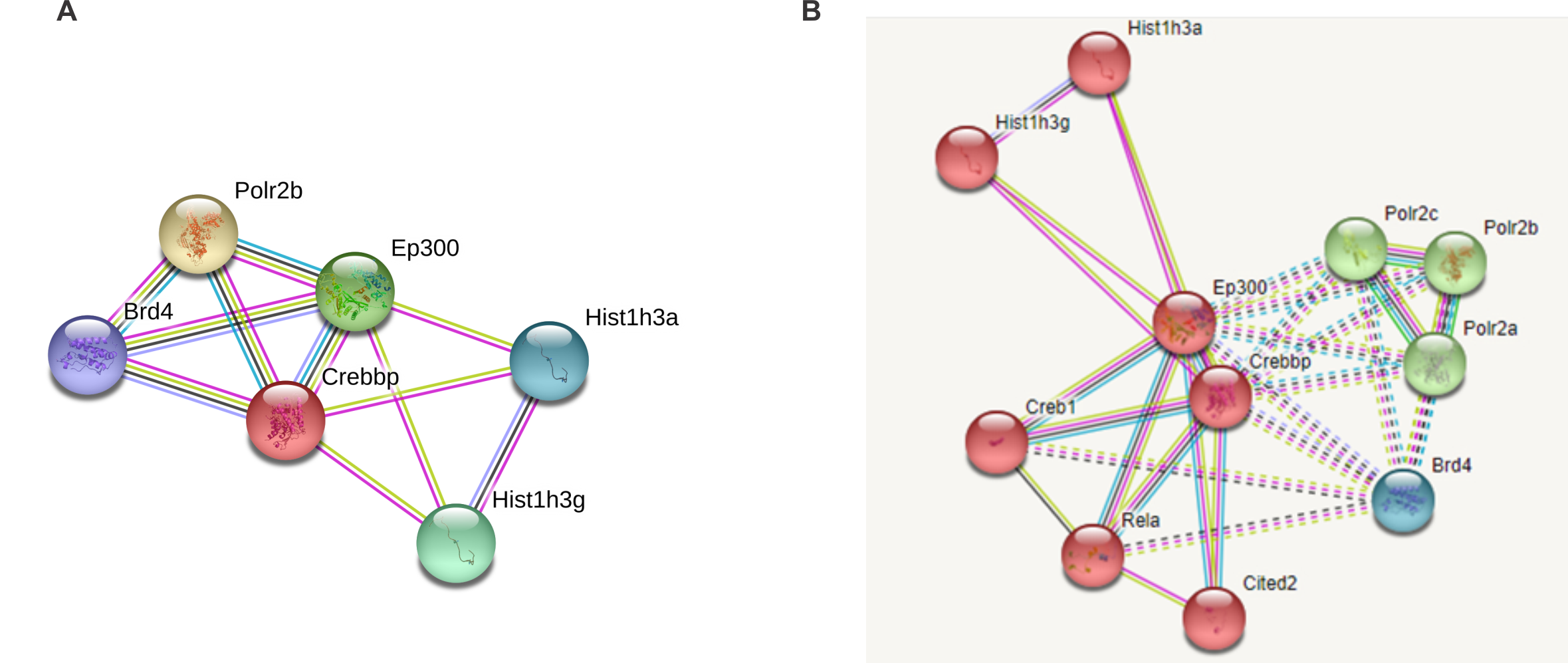

Supplement: S1 Fig — (A) Protein-protein interaction prediction among BRD4, p300, RNA pol II and Histone H3 by STRING V.11. (B) Classification of interacting partners of BRD4 into different groups based on the interacting protein functions. (TIF) [file ppat.1009343.s001.tif]

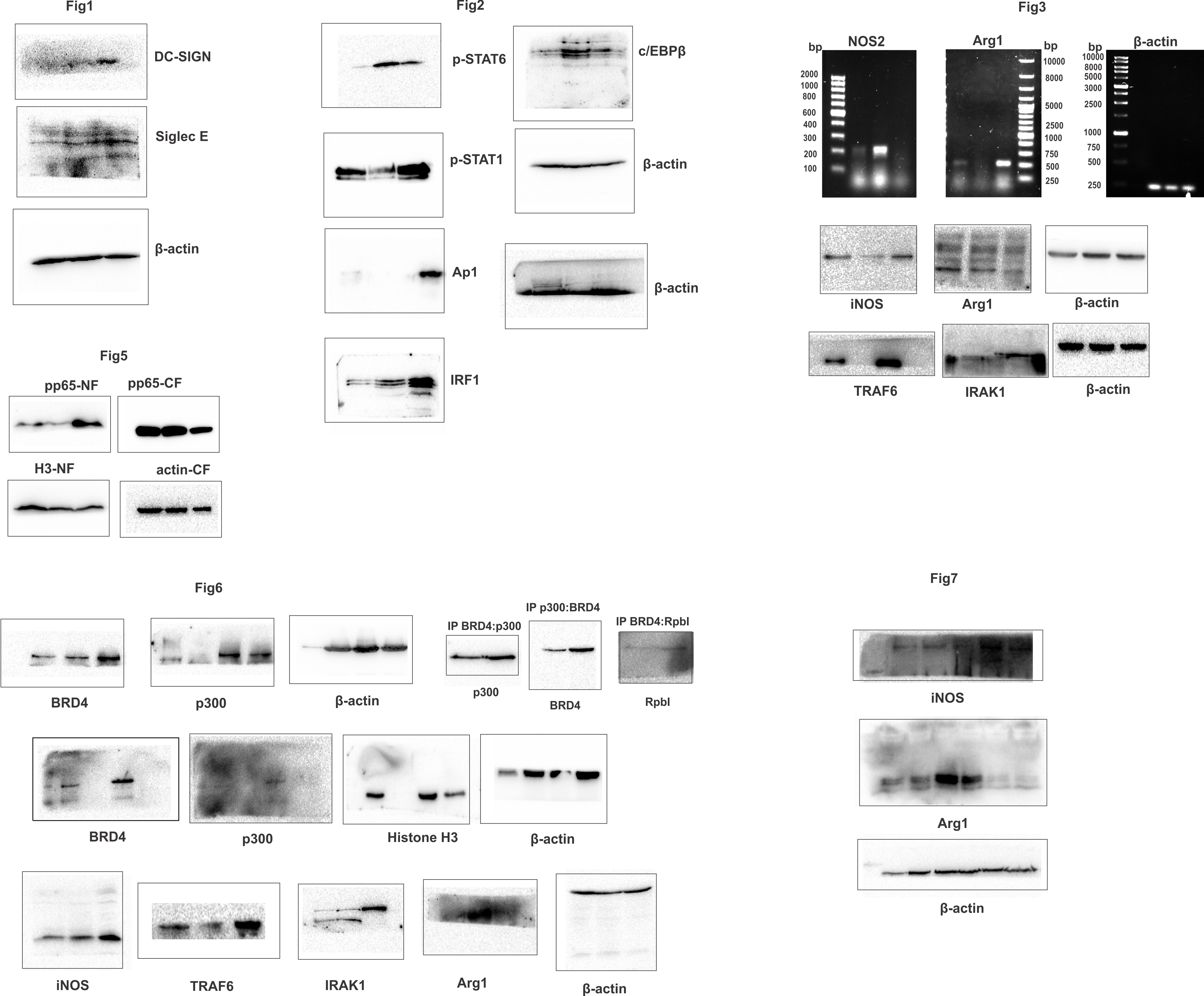

Supplement: S2 Fig — (TIF) [file ppat.1009343.s002.tif]
