# Supplementary material for: Super enhancer-mediated transcription of miR146a-5p drives M2 polarization during Leishmania donovani infection
Source: PLoS Pathog. 2021 Feb 25;17(2):e1009343. doi: 10.1371/journal.ppat.1009343 (PMC7943006; doi:10.1371/journal.ppat.1009343)
Supplement: S1 Table — (DOCX) [file ppat.1009343.s003.docx]

| Sl. No | Gene name | Primer sequence |  |
| --- | --- | --- | --- |
| 1 | YM-1 | 5’GGGCATACCTTTATCCTGAG 3’ | Forward |
|  |  | 5’CCACTGAAGTCATCCATGTC 3’ | Reverse |
| 2 | FIZZ1 | 5'- TCCCAGTGAATACTGATGAGA-3' | Forward |
|  |  | 5'-CCACTCTGGATCTCCCAAGA-3' | Reverse |
| 3 | CCR7 | 5′-TCATTGCCGTGGTGGTAGTCTTCA-3′ | Forward |
|  |  | 5′-ATGTTGAGCTGCTTGCTGGTTTCG-3′ | Reverse |
| 4 | Arg-1 | 5'-CAGAAGAATGGAAGAGTCAG-3' | Forward |
|  |  | 5'- CAGATATGCAGGGAGTCACC -3' | Reverse |
| 5 | β-Actin | 5'- ACACTGTGCCCATCTACGAG-3' | Forward |
|  |  | 5'-TCAACGTCACACTTCATGATG-3' | Reverse |
| 6 | TRAF6 | 5'-ATTTCATTGTCAACTGGGCA-3' | Forward |
|  |  | 5'-TGAGTGTCCCATCTGCTTGA-3' | Reverse |
| 7 | IRAK1 | 5'-GAGACCCTTGCTGGTCAGAG-3' | Forward |
|  |  | 5'-GCTACACCCACCCACAGAGT-3' | Reverse |
| 8 | miR 146a-5p(assay ID- 478399_mir) | Mature miRNA seq-UGAGAACUGAAUUCCAUGGGUU | Taqman probed |
| 9 | U6 snRNA | Assay id- Hs01123609_g1 |  |
| 10 | miR146a-5p enhancer | GTCTTGCTGAGGAGGTG | Forward |
| 11 | miR146a-5p enhancer | AGACGAGCTGCTTCAAGT | Reverse |

**Table S1.** List of primers used for real time and semi quantitative PCR reactions
